# Noncanonical functions of UGT2B17 promote castration-resistant prostate cancer progression

**DOI:** 10.1172/JCI196495

**Published:** 2025-12-04

**Authors:** Tingting Feng, Ning Xie, Lin Gao, Qiongqiong Jia, Sonia H.Y. Kung, Tunc Morova, Yinan Li, Lin Wang, Ladan Fazli, Louis Lacombe, Chantal Guillemette, Eric Lévesque, Nathan A. Lack, Jianfei Qi, Bo Han, Xuesen Dong

**Affiliations:** 1 Biomedical Sciences College & Shandong Medicinal Biotechnology Centre, Shandong First Medical University & Shandong Academy of Medical Sciences, Jinan, Shandong, China.; 2 The Vancouver Prostate Centre, Vancouver General Hospital, Vancouver, British Columbia, Canada.; 3 Department of Pathology, School of Basic Medical Sciences, Shandong University, Jinan, China.; 4 Department of Vascular Surgery, Renji Hospital, Shanghai Jiao Tong University School of Medicine, Shanghai, China.; 5 Faculty of Medicine, and; 6 Faculty of Pharmacy, Centre de recherche du Centre Hospitalier Universitaire de Québec - Université Laval (CRCHUQc-UL), Québec, Québec, Canada.; 7 Centre de recherche du Centre Hospitalier Universitaire de Québec - Université Laval (CRCHUQc-UL), Centre de recherche sur le cancer (CRC) de l’Université Laval, Faculty of Medicine, Université Laval, Québec, Québec, Canada.; 8 Department of Urologic Sciences, University of British Columbia, Vancouver, British Columbia, Canada.; 9 Department of Medical Pharmacology, School of Medicine, Koc University, Istanbul, Turkey.; 10 Department of Biochemistry and Molecular Biology, University of Maryland, Baltimore, Maryland, USA.; 11 Department of Pathology, Peking University People’s Hospital, Beijing, China.

**Keywords:** Clinical Research, Oncology, Cell stress, Prostate cancer, Protein misfolding

## Abstract

Androgen deprivation therapy is the primary treatment for advanced prostate tumors. While initially effective, tumor progression to the therapy-resistant stage is inevitable. Paradoxically, UDP glucuronosyltransferase family 2 member B17 (UGT2B17), the key enzyme responsible for androgen catabolism in prostate tumor cells, is upregulated in therapy-resistant tumors, though its role in tumor progression remains unclear. Here, we demonstrate that UGT2B17 possesses multiple oncogenic functions independent of androgen catabolism. It modulates protein-folding pathways, allowing tumor cells to endure therapy-induced stress. UGT2B17 also regulates transcription associated with cell division and the DNA damage response, enabling unchecked cell proliferation. Targeting the newly identified UGT2B17 functions using a combination of inhibitors reduced tumor growth in therapy-resistant tumor models, highlighting a promising therapeutic strategy. Collectively, these findings reveal a mechanism by which prostate tumors exploit UGT2B17 to evade therapy and highlight its potential as a therapeutic target in advanced prostate cancer.

## Abstract 2

UGT2B17’s non-enzymatic function facilitates prostate cancer cells to resist therapy-induced stress and promote tumor progression.

## Graphical abstract

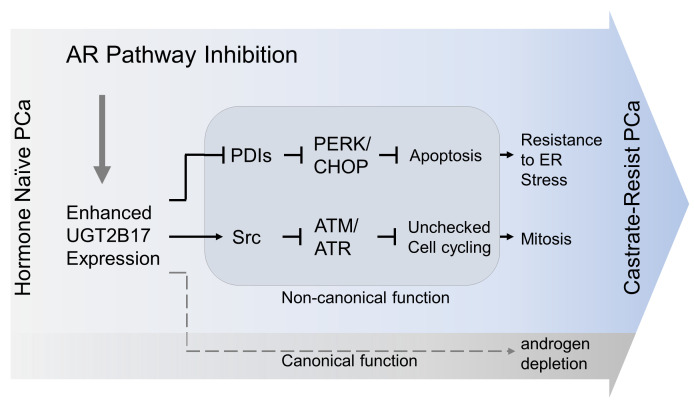

## Introduction

Prostate cancer (PCa) is the most commonly diagnosed cancer in men and a leading cause of cancer-related deaths globally. Current therapies for metastatic PCa center on androgen pathway inhibition, by either suppressing androgen synthesis or preventing androgens from activating androgen receptor (AR) (1, 2). Despite initial efficacy, resistance invariably arises, resulting in lethal tumor progression to castration-resistant prostate cancer (CRPC) stage. Although detectable androgens and androgenesis enzymes were reported in CRPC tissue homogenates (3–5), it remains unclear whether interstitial androgens in CRPC are required to drive tumor growth. This question is important for oncologists to decide whether antiandrogens should be continuously given to a patient.

Within PCa cells, the bioavailability of androgens to activate AR is determined by both androgenesis and androgen catabolism. UDP glucuronosyltransferase family 2 member B17 (UGT2B17) is the primary androgen catabolic enzyme in PCa cells, conjugating uridine diphosphate glucuronic acid (UDPGA) to dihydrotestosterone (DHT) and testosterone (T). This reaction increases androgen solubility, reduces AR affinity, and accelerates excretion (6). UGT2B17 contains a substrate-binding domain at its N-terminus that binds androgens and a cofactor-binding domain at its C-terminus that facilitates UDPGA conjugation (7). It is a highly efficient androgen catabolic enzyme, enabling rapid androgen clearance (8). These findings suggested that increased UGT2B17 expression in PCa cells can deplete intracellular androgens effectively, even in the presence of increased androgenesis by PCa cells and surrounding stromal cells.

Indeed, UGT2B17 expression is regulated by PCa cells through a reciprocal feedback mechanism, whereby antiandrogens upregulate UGT2B17 expression (7, 9) to accelerate androgen clearance from PCa cells. Conversely, DHT suppresses UGT2B17 expression to preserve intracellular androgen levels (8, 10). This mutual regulation presents a paradox that androgen pathway inhibition and its consequential UGT2B17 upregulation should, theoretically, eliminate androgens completely in PCa cells, leading to irreversible tumor suppression and no CRPC progression, if the tumors rely on androgens to progress. However, the inevitability of CRPC suggests that UGT2B17 may have additional non-androgen catabolic functions that facilitate PCa cell survival and promote CRPC progression.

Here, we report that UGT2B17 has tumor-promoting functions beyond androgen catabolism. UGT2B17 interacts with proteins involved in the unfolded protein response (UPR), enabling PCa cells to resist endoplasmic reticulum (ER) stress. Additionally, UGT2B17 regulates transcription associated with mitosis and DNA damage response (DDR), primarily through Src kinase, deactivating ataxia telangiectasia and Rad3-related protein (ATR) and ataxia-telangiectasia mutated (ATM) kinases to bypass G2/M checkpoints to accelerate cell proliferation. These findings suggest that while blocking androgen signaling increases UGT2B17 expression to reduce the androgen dependency of PCa cells, UGT2B17 simultaneously exerts non-androgen catabolic functions to support PCa cell survival and growth, ultimately promoting CRPC progression.

## Results

### UGT2B17 protein expression in patients with PCa.

**Figure 1:**
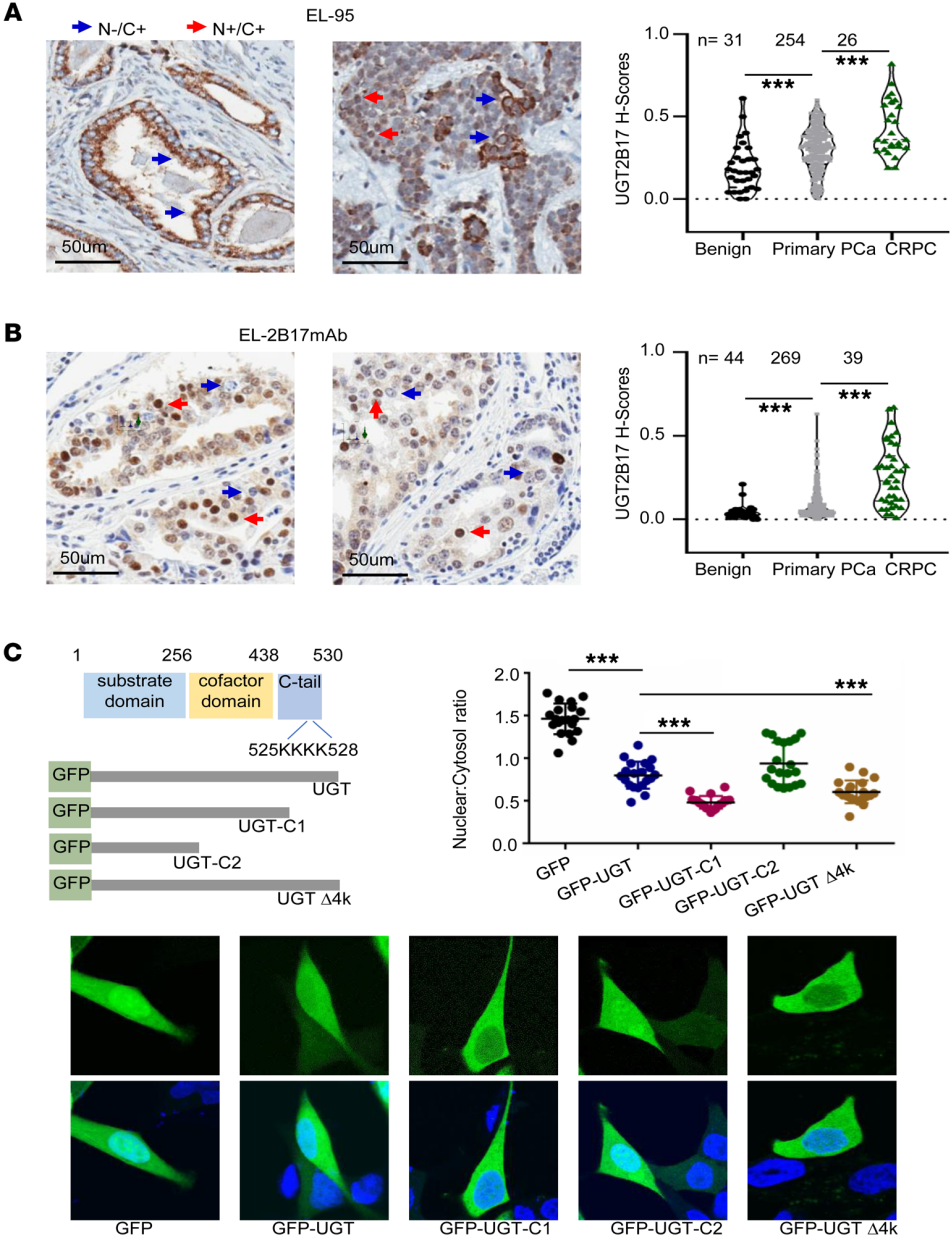
UGT2B17 protein expression is upregulated in CRPC patient tumors. (**A** and **B**) Tissue microarrays containing PCa tissue cores were stained with 2 UGT2B17 antibodies, EL-95 (**A**) and EL-2B17mAb (**B**). Pathology scoring of UGT2B17 protein expression by each antibody was plotted. Blue arrows: nuclear localization is negative while cytoplasmic localization is positive. Red arrows: both cytoplasmic and nuclear localizations are positive. (**C**) Vectors expressing EGFP-tagged UGT2B17 (aa1–530) and mutant UGT2B17 (C1, aa1–438; C2, aa1–256; Δ4, deletion of aa525–528) were transfected in LNCaP cells and subjected to confocal microscopy. Twenty cells from random areas of each slide were used to quantify the GFP signal from the cytosol and nuclei by ImageJ (NIH). Experiments were performed 3 times with similar results obtained. N-/C+ means negative nuclear staining and positive cytoplasm staining. N+/C+ means positive staining in both nucleus and cytoplasm. Data are shown as the mean ± SEM. Statistical tests performed by 1-way ANOVA test. ***P < 0.001.

To determine UGT2B17 protein expression during CRPC progression, we utilized 2 previously validated IHC-grade antibodies, EL-95 and EL-2B17mAb (11–13). Both antibodies generated nuclear and cytoplasmic signals, with EL-95 predominantly exhibiting cytoplasmic signals and EL-2B17mAb favoring nuclear signals (Figure 1, A and B; Supplemental Figure 1; and Supplemental Figure 2A; supplemental material available online with this article; https://doi.org/10.1172/JCI196495DS1). These differences reflect the distinct antigens used to develop these antibodies and the various conformations that UGT2B17 may adopt in different subcellular locations. Consistent with previous studies, our histological evaluation of UGT2B17 in the cytoplasm and nucleus separately revealed the same trend of change. Therefore, we scored the total UGT2B17 signal using Aperio ImageScope and found that UGT2B17 expression was 1.5- to 2.8-fold higher in primary prostate tumors compared with benign prostate tissue and 1.4- to 3-fold higher in CRPC than in primary tumors (Figure 1, A and B). Importantly, these results are consistent with findings from an independent study involving 2 large patient cohorts, the Canadian Prostate Cancer Biomarker Network biobank (*n* = 1,454) and the PROCURE cohort (*n* = 1,562), in which elevated UGT2B17 protein expression was reported to be associated with an increased risk of CRPC progression and prostate cancer–specific mortality (14). Together, these results implied that upregulation of UGT2B17 promotes CRPC progression.

The subcellular localization patterns of UGT2B17 suggest its roles extend beyond androgen catabolism, since the glucuronosyltransferase activity of UGT2B17 is confined to the ER lumen, where the cofactor of UDPGA is located (7, 15). Although UGT2B17 is deemed to have a ER retention signal peptide of 519-KLAKTGKKKKRD-530″ at its C-terminus (7), predictions from NLStradamus (16) and other algorithms suggest that this sequence also functions as a nuclear localization signal (NLS), overlapping with the ER retention signal. Our confocal microscopy verified that GFP-tagged UGT2B17 is primarily localized in the cytoplasm but also in the nucleus (Figure 1C). To rule out the possibility that GFP cleavage from the N-terminus of the GFP-UGT2B17 chimer protein could lead to inaccurate subcellular localization, we constructed vectors expressing UGT2B17 and a C-terminal Flag tag (Supplemental Figure 2B). Immunofluorescence (IF) assays using a Flag antibody showed similar results with those obtained using the GFP-UGT2B17 constructs, confirming that UGT2B17 localizes to multiple subcellular compartments. Depletion of the C-terminal region (UGT-C1) or mutation of the 4 lysine residues (UGTΔ4) reduced the nuclear localization of UGT2B17 (Figure 1C and Supplemental Figure 2B). The dynamic shuttling of UGT2B17 between the cytoplasm and nucleus was also observed by the decreased nuclear-to-cytoplasmic ratio of UGT2B17 following extracellular stimuli, such as dasatinib (Supplemental Figure 2C). Furthermore, nuclear and cytoplasmic fractionation combined with immunoblotting assays confirmed that UGT2B17 is localized in both compartments (Supplemental Figure 2D). Collectively, results from IHC, confocal microscopy, and cellular fractionation assays all confirmed that UGT2B17 is present in multiple subcellular compartments, suggesting potential non-androgen catabolic functions.

### Prolonged androgen deprivation enhances UGT2B17 protein stability in PCa cells.

The mechanism underlying UGT2B17 upregulation in CRPC remains unclear. AR was reported to be recruited to the UGT2B17 promoter, suppressing UGT2B17 transcription in androgen-sensitive PCa cells treated with DHT for 24–48 hours (10). However, this mechanism does not fully explain the elevated UGT2B17 expression in CRPC, as the AR signaling in many tumors is restored following castration therapy. We also observed that 2 CRPC cell models, LNCaP95 and MR49F, derived from LNCaP cells through long-term androgen depletion or enzalutamide treatment (17), express higher UGT2B17 protein than LNCaP cells (Supplemental Figure 3A). However, UGT2B17 mRNA levels in MR49F cells remained low, similar to those in LNCaP cells, suggesting that posttranscriptional mechanisms contribute to UGT2B17 upregulation. To support this hypothesis, we found that UGT2B17 protein stability was enhanced in LNCaP cells stably overexpressing UGT2B17 (designated LNCaP [UGT]) after they were androgen-depleted for 28 days (Supplemental Figure 3B). Similarly, MR49F cells exhibited greater UGT2B17 protein stability compared with parental LNCaP cells. Our in vitro ubiquitination assays and proximity ligation assays (PLAs) confirmed that 28 days of androgen depletion reduced UGT2B17 ubiquitination (Supplemental Figure 3, C and D). Deletion mutagenesis assays further revealed that at least 2 domains, the C-terminal tail and the cofactor-binding domain of UGT2B17, were targeted by the ubiquitin-proteasome pathway (Supplemental Figure 3E). Together, these findings confirm that prolonged androgen deprivation enhances UGT2B17 protein stability in PCa cells, consistent with increased UGT2B17 expression during CRPC progression (Figure 1) to exert non-androgen catabolic functions.

### UGT2B17 regulates UPR to counteract ER stress.

**Figure 2:**
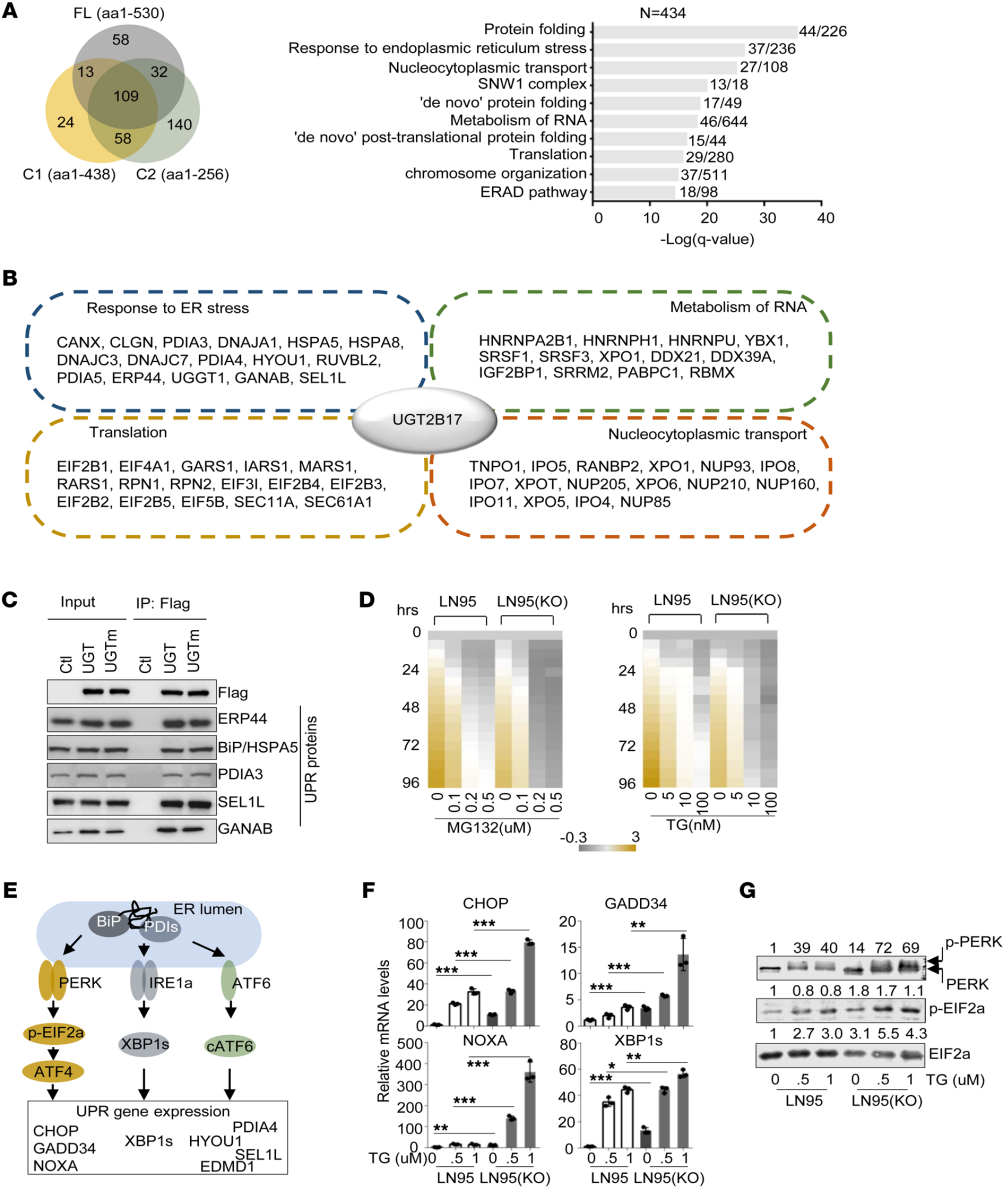
UGT2B17 regulates UPR in PCa cells. (**A**) LNCaP cells transfected with Flag-tagged control, UGT2B17, C1, or C2 constructs were subjected to co-IP with a Flag antibody, followed by MS analysis (n = 4 repeats/co-IP plus MS). Proteins bound to Flag-UGT2B17, Flag-C1, and Flag-C2, compared with Flag-control (with unique peptides > 3 and abundance > 100,000), were analyzed using Metascape. The top ranked gene annotations were ranked by –log_10_(*q* value) and plotted. (**B**) Selected proteins within the annotation groups from **A** are listed. (**C**) Co-IP of Flag-tagged UGT2B17 or UGTm with proteins identified from **A**. (**D**) LN95 and LN95(KO) cells were treated with increasing doses of TG and MG for 0–96 hours as indicated. Cell viability was measured by Incucyte and plotted over control at day 0. (**E**) A diagram summarizes 3 UPR pathways and their downstream effectors. (**F**) LNCaP95 and LN95(KO) cells were treated with increasing doses of TG for 24 hours, and mRNA levels of UPR genes were measured by real-time qPCR. (**G**) LNCaP95 and LN95(KO) cells were treated with increasing doses of TG for 24 hours, and protein levels of PERK and EIF2a and their phosphorylated forms were measured by immunoblotting. Densitometry of protein bands were quantified by ImageJ and normalized to that from vehicle treatment conditions. Experiments in **C**, **D**, **F**, and **G** were performed 3 times with similar results obtained. Data are shown as the mean ± SEM. Statistical tests performed by 2-way ANOVA test. *P < 0.05, **P < 0.01, ***P < 0.001.

To explore the non-androgen catabolic activities of UGT2B17 globally, we conducted affinity purification combined with mass spectrometry (MS) using Flag-tagged UGT2B17 and its deletion mutants, C1 and C2, transfected in LNCaP cells. UGT2B17-associated proteins were identified by MS. A total of 212, 204, and 339 proteins (unique peptides > 3) from either cytoplasmic or nuclear fractions of the cells interacted with UGT2B17, C1, and C2, respectively, with 109 proteins shared among all UGT2B17 isoforms (Figure 2A and Supplemental Data 1). Metascape analyses (18) revealed that these proteins clustered into similar functional groups, implying that each domain of UGT2B17 interacts with protein components of the same complexes. The top ranked gene annotations included protein folding, response to ER stress, translation, nucleocytoplasmic transport, and metabolism of RNA. Representative proteins in each annotation group were listed (Figure 2B). These results highlighted several key insights: 1) UGT2B17 interacts with both cytoplasmic and nuclear proteins, indicating its involvement in diverse cellular processes beyond androgen catabolism, which is confined to the ER lumen; 2) UGT2B17 associates with nucleocytoplasmic transport proteins, suggesting its dynamic trafficking across subcellular compartments to mediate diverse cytoplasmic and nuclear functions; and 3) UGT2B17 interacts with protein complexes involved in RNA processing/pre-mRNA splicing, translation, and protein folding, implying UGT2B17’s role in regulating protein expression through multiple posttranscriptional mechanisms. These results corroborate our IHC findings from PCa patient biopsies and support a non-androgen catabolic function for UGT2B17.

We focused on UGT2B17-associated proteins involved in protein folding, response to ER stress, and translation, as balanced mRNA translation in the cytosol and proper protein folding inside the ER lumen are essential for cell viability. Cancer cells exhibit a greater capacity than benign cells to manage ER stress caused by hypoxia, radiation, and chemotherapy (19). The accumulation of misfolded proteins in the ER is cytotoxic, prompting cancer cells to activate UPR pathways mediated by inositol-requiring enzyme 1a (IRE1a), activating transcription factor 6 (ATF6), and protein kinase RNA-like endoplasmic reticulum kinase (PERK) to mitigate ER stress (19). To validate our MS findings, we conducted co-immunoprecipitation (co-IP) of at least 5 UPR proteins (ERP44, BiP, PDIA3, SEL1L, and GANAB), confirming their association with UGT2B17 (Figure 2C). Notably, these UPR proteins remain associated with the UGT2B17 mutant (UGTm), which lacks androgen catabolic activity because of S121Y, N124S, and I125N mutations (20). These results demonstrate that UGT2B17 interactions with UPR proteins are independent of androgen catabolism, highlighting its non-androgen catabolism functions.

To test whether UGT2B17 regulates UPR to counteract ER stress, we treated LNCaP95 cells and UGT2B17-knockout [LN95(KO)] cells (Supplemental Figure 4A) with 2 ER stress inducers, thapsigargin (TG) and MG132 (MG). LNCaP95 cells tolerated 0.1 μM MG and 5–10 nM TG treatments, maintaining growth over 96 hours (Figure 2D). At higher concentrations (0.2–0.5 μM MG and 100 nM TG), their viability was initially suppressed at 24 hours but recovered after 48 hours. In contrast, LN95(KO) cells exhibited reduced viability at 0.2–0.5 μM MG and 10–100 nM TG, indicating that UGT2B17 enhances PCa cell resistance to ER stress. To determine which UPR pathways are regulated by UGT2B17, we treated LNCaP95 and LN95(KO) cells with increasing doses of TG. Depletion of UGT2B17 primarily induced hyperactivation of the PERK pathway (Figure 2, E and F), as evidenced by increased mRNA levels of CHOP, GADD34, and NOXA, along with elevated phospho-PERK and phospho-EIF2α protein levels (Figure 2G). A mild increase in IRE1a pathway activation was observed, as indicated by increased XBP1s mRNA levels (Figure 2F), whereas the ATF6 pathway remained unaffected (Supplemental Figure 4B). Notably, basal levels of PERK and IRE1a signaling in LN95(KO) cells were higher than those in LNCaP95 cells (Figure 2, F and G), which is consistent with the reduced viability of LN95(KO) cells compared with LNCaP95 cells (Figure 2D). These results suggest that UGT2B17 provides a protective function in PCa cells and that its loss leads to defects in protein folding and overactive ER stress, even in the absence of stress inducers. While UPR activation generally promotes cell survival, excessive UPR triggers cell cycle arrest and apoptosis, acting as a safeguard against irreversibly damaged cells.

Together, these results demonstrate that UGT2B17 mainly regulates the PERK pathway, which is crucial for determining PCa cell viability under toxic ER stress conditions.

### Non-androgen catabolic activity of UGT2B17 regulates CHOP-mediated apoptosis.

**Figure 3:**
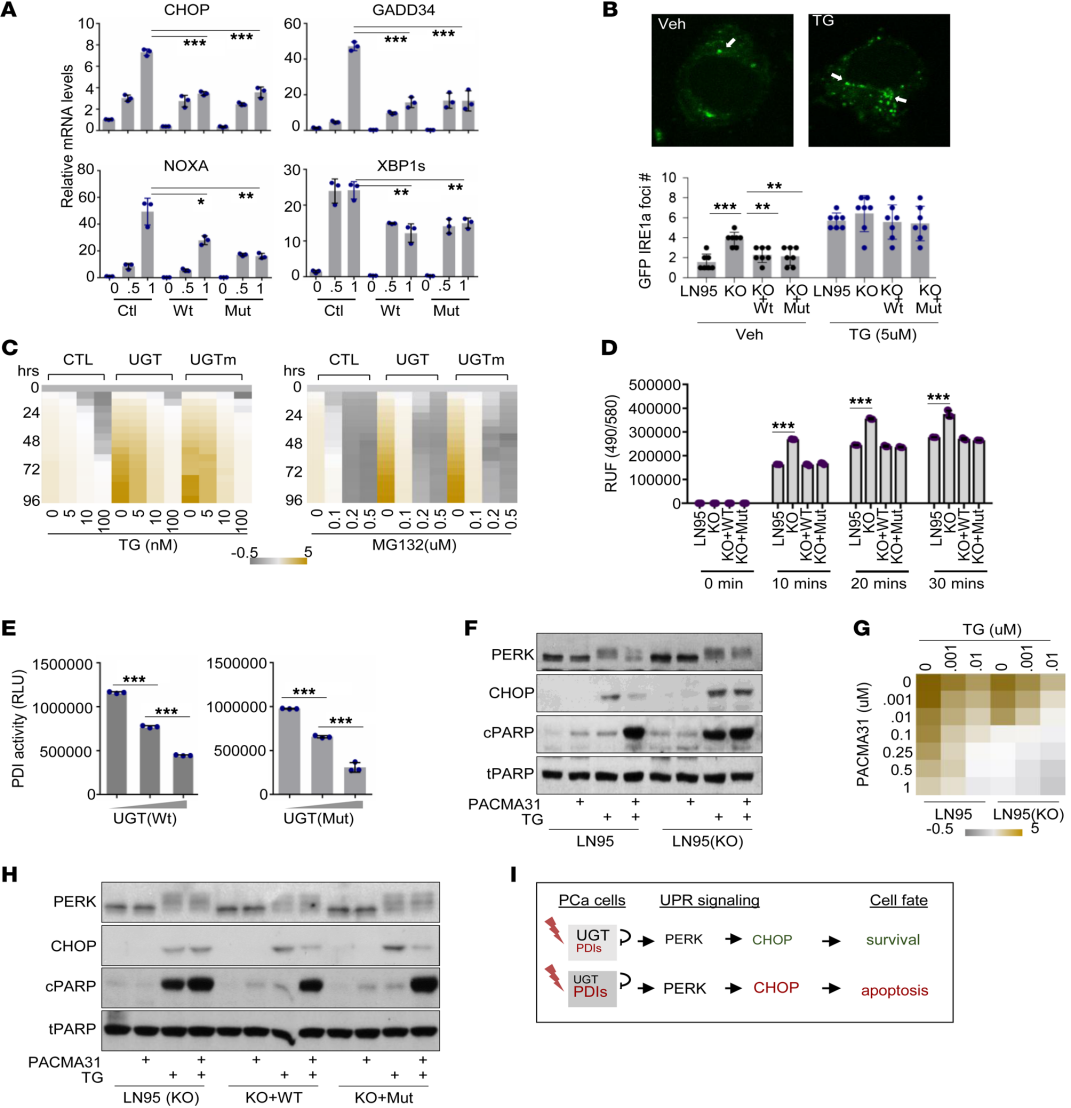
UGT2B17 regulates PERK and IRE1a pathways independent of its androgen-catabolic activity. (**A**) LN95(KO) cells were transfected with control, UGT2B17, and UGTm for 16 hours. Real-time qPCR measured the mRNA levels of the indicated genes. (**B**) LNCaP cells were transfected with GFP-tagged IRE1a and treated with vehicle or 5 μM TG for the 6 hours. IRE1a oligomerization was monitored by confocal microscopy and counted in 7 randomly selected high-power fields. (**C**) LN95(KO) cells were transfected with control, UGT2B17, or UGTm for 16 hours. Cells were then treated with increasing doses of TG or MG for 0–96 hours. Cell viability was measured by Incucyte and plotted over vehicle treatment at day 0. (**D**) Protein lysates were collected from LNCaP95, LN95(KO), and LN95(KO) cells transfected with UGT2B17 or UGTm for 72 hours. The PDI activity was measured. (**E**) Purified Flag UGT2B17 and UGTm were used to measure their impacts to PDI activity. (**F**) LNCaP95 and LN95(KO) cells were treated with 2 μm PACMA31 and/or 1 μM TG for 16 hours. Cell lysates were collected for immunoblotting using the indicated antibodies. (**G**) LNCaP95 and LN95(KO) cells were treated with increasing doses of PACMA31 and TG for 16 hours. Cell viability was measured and plotted over vehicle treatment. (**H**) LN95(KO) cells transfected with UGT2B17 or UGTm were treated with 2 μm PACMA31 and/or 1 μM TG for 16 hours. Cell lysates were collected for immunoblotting using the indicated antibodies. (**I**) A model depicting PDI activity suppressed by UGT2B17 to control the PERK/CHOP pathway to determine the cell fate. Experiments in **A**–**H** were performed 3 times with similar results obtained. Data are shown as the mean ± SEM. Statistical tests performed by 2-way ANOVA test (**A**, **B**, and **D**), 1-way ANOVA test (**E**). **P < 0.01, ***P < 0.001.

To further demonstrate whether UGT2B17 relies on its androgen catabolic activity to regulate the PERK pathway, we transfected LN95(KO) cells with either UGT2B17 or UGTm. Both UGT2B17 proteins inhibited the mRNA levels of CHOP, GADD34, and NOXA (Figure 3A) but not PDIA4, SEL1L, HYOU1, and EDMD1 (Supplemental Figure 4C). Similar results were also obtained from PC3, DU145, and 22Rv1 cell models transfected with either UGT2B17 or UGTm (Supplemental Figure 5). The androgen catabolic activity of UGT2B17 was not required to regulate the IRE1a pathway, as evidenced by the mRNA levels of XBP1s (Figure 3A and Supplemental Figure 5) and the rates of IRE1a oligomerization in UGT2B17- and UGTm-transfected cells (Figure 3B). Furthermore, both UGT2B17 and UGTm enhanced the viability of PCa cells under ER stress conditions (Figure 3C), confirming that UGT2B17 regulates the UPR to cope with ER stress, and this action is independent of its androgen catabolic activity.

While PERK and IRE1a are sensors of ER stress, it remains unclear how UGT2B17 regulates the UPR to promote cell survival, rather than allowing it to become hyperactivated and trigger apoptosis. UGT2B17 interacts with several protein disulfide isomerases (PDIs) (Figure 2), which have dual roles in maintaining cell survival or inducing apoptosis. PDIs promote protein folding by catalyzing the isomerization, reduction, and oxidation of disulfides (21) to counteract ER stress and maintain cell viability. In contrast, hyperactive PDI induces Bak oligomerization in mitochondria and increases mitochondrial outer membrane permeabilization, triggering apoptosis (22), an action that can be suppressed by PDI inhibitors (23). These findings suggest that UGT2B17 may be a critical modulator of PDIs to control PCa cell viability. There are 21 PDI members reported in human cells, and previous published RNA sequencing results indicated that the mRNAs of several PDIs are highly expressed in all cell models, with PDIA1 (also known as P4HB) and PDIA3 (also known as ERP57) being the most abundantly expressed (Supplemental Figure 6A). Both were found to interact with UGT2B17 (Figure 2). We confirmed that neither UGT2B17, UGTm, nor DHT treatment altered the protein levels of PDIA1, PDIA3, and ERP44, suggesting that UGT2B17 may regulate the enzyme activity of these PDIs (Supplemental Figure 6, B and C).

To test this hypothesis, we collected protein lysates from LNCaP95 and LN95(KO) cells and found that UGT2B17 depletion resulted in enhanced PDI activities (Figure 3D). Conversely, lysates from LN95(KO) cells transfected with UGT2B17 and UGTm showed inhibitory effects on PDI activity. Since cell lysates contain many proteins that could be altered by UGT2B17, potentially influencing PDI activity indirectly, we purified Flag-tagged UGT2B17 and UGTm proteins from HEK293T cells and found that both proteins suppressed PDIA1 activity in vitro in a dose-dependent manner (Figure 3E). We also confirmed by using protein pulldown assays that both UGT2B17 and UGTm interacted with PDIA3 and PDIA1 directly (Supplemental Figure 6, D and E). Given that UGT2B17 depletion reduced PCa cell viability, these results indicate that UGT2B17 prevents overactive PDIs from triggering apoptosis. To further test this, we challenged LNCaP95 cells with TG alone or in combination with PACMA31, a PDI inhibitor. We found that LNCaP95 cells could cope with TG treatment by activating PERK/CHOP signaling, resulting in minimal cell apoptosis, as reflected by cPARP levels (Figure 3F). This protective action of UGT2B17 relied on PDI activity, since cotreatment with PACMA31 reduced PERK/CHOP signaling, leading to extensive cell apoptosis. In contrast, LN95(KO) cells responded to TG or TG plus PACMA31 with strong PERK and CHOP expression, reflecting that the cells underwent apoptosis. These results indicated that UGT2B17 loss leads to the irreversible accumulation of misfolded proteins and hyperactive PDIs that cannot be rescued by PACMA31, resulting in the constitutive activation of PERK/CHOP signaling and cell apoptosis.

These molecular changes were consistent with the observed PCa cell viability, as UGT2B17 conferred stronger viability to PCa cells even under TG or PACMA31 treatment (Figure 3G). Additionally, we confirmed that the PDI activity regulated by UGT2B17 is independent of its androgen catabolic activity, since both UGT2B17 and UGTm prevented LN95(KO) cells from undergoing apoptosis under TG treatment (Figure 3H). Based on these results, we propose a model in which UGT2B17 suppresses overactive PDIs in PCa cells under ER stress conditions, allowing the PERK/CHOP signaling pathway to maintain cell viability (Figure 3I). Loss of UGT2B17 results in hyperactive PDI under stress conditions, leading to CHOP-induced cell apoptosis.

### UGT2B17 regulates gene transcription associated with mitosis and DNA damage response.

**Figure 4:**
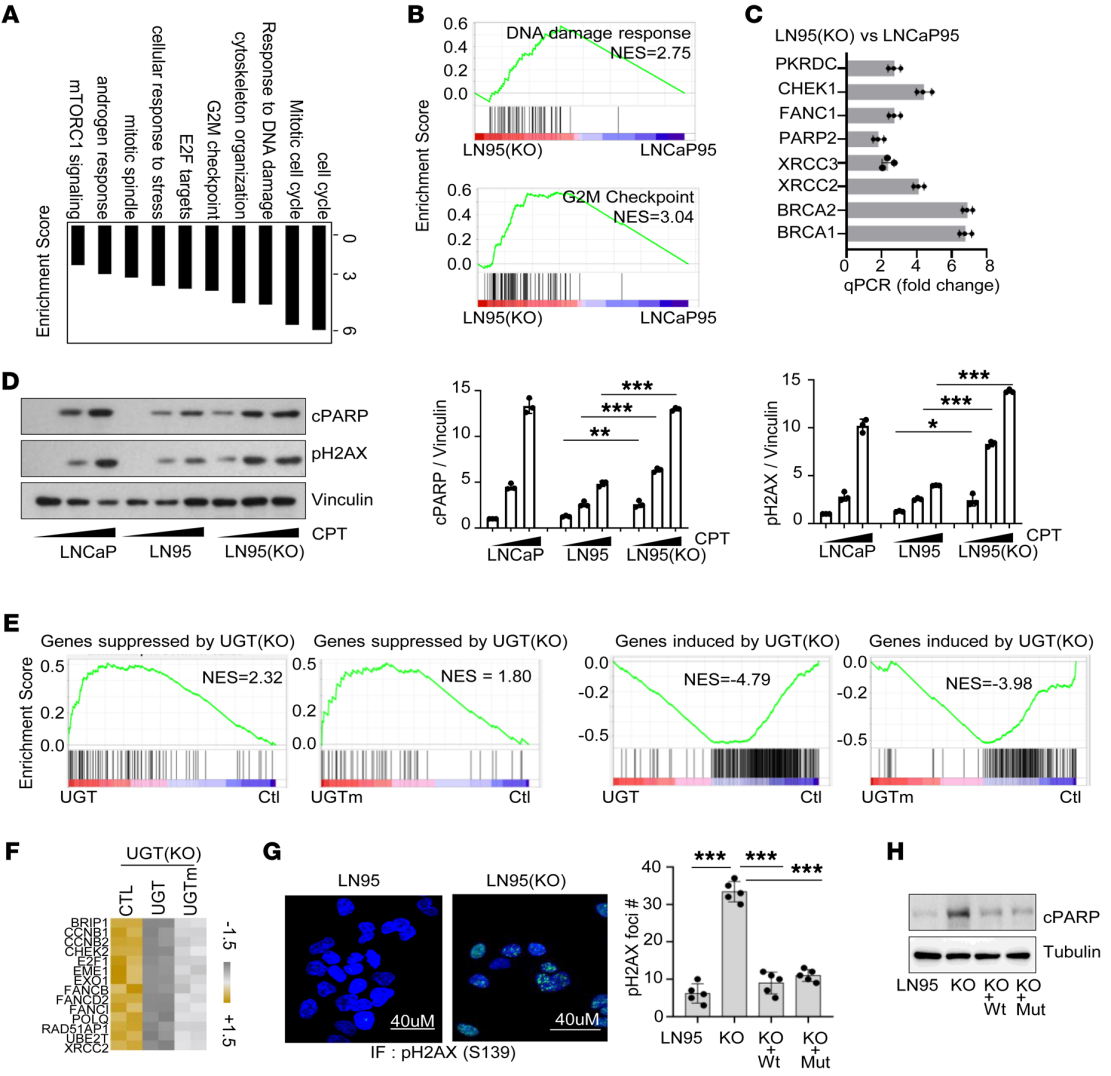
UGT2B17 regulates transcription associated with mitosis and DDR. (**A** and **B**) RNA-Seq analysis comparing the transcriptome between LNCaP95 and LN95(KO) cells (*n* = 2 repeats). GSEA showed top ranked signal pathways regulated by UGT2B17 knockout. (**C**) Real-time qPCR validated 8 DDR genes altered by UGT2B17 knockout in LNCaP95 cells. (**D**) LNCaP, LNCaP95, and LN95(KO) cells were treated with increasing doses of CPT. Indicated protein markers were measured by immunoblotting. Densitometry of protein bands from triplicate experiments was determined by ImageJ and plotted. (**E**) LN95(KO) cells were transfected with control, UGT2B17, or UGTm vector and subjected to RNA-Seq analysis. GSEA tested the association between the gene sets altered by UGT2B17 or UGTm and the gene sets altered by UGT2B17 knockout in the LNCaP95 cells. (**F**) A heatmap shows RNA-Seq data of genes associated with cell mitosis and DDR in LN95(KO) cells overexpressing either UGT2B17 or UGTm. (**G**) LNCaP95, LN95(KO), and LN95(KO) cells overexpressing UGT2B17 or UGTm vector were used to measure the number of p-H2AX foci in 5 randomly selected high-power fields. (**H**) LNCaP95, LN95(KO), and LN95(KO) cells overexpressing UGT2B17 or UGTm were used to measure the apoptosis marker of cPARP. Experiments in **C**, **D**, **G**, and **H** were performed 3 times with similar results obtained. Data are shown as the mean ± SEM. Statistical tests performed by 2-way ANOVA test (**D**), 1-way ANOVA test (**G**). *P < 0.05, **P < 0.01, ***P < 0.001.

To investigate if UGT2B17 exerts any nuclear functions, we performed RNA-Seq analysis comparing the transcriptomes of LNCaP95 and LN95(KO) cells. We identified 1,546 gene alterations (fold-change > 2, and adj *P* < 0.05) regulated by UGT2B17 (Supplemental Figure 7A and Supplemental Data 2). Gene set enrichment analysis (GSEA) showed that the top ranked pathways regulated by UGT2B17 were cell cycling/cell mitosis, G2/M checkpoint, and DNA damage response (DDR) (Figure 4, A and B). Among the differentially expressed genes, at least 54 were associated with DDR, and 8 of these were validated by real-time qPCR analyses (Figure 4C). Consistently, when LNCaP, LNCaP95, and LN95(KO) cells were treated with the DNA damage inducer camptothecin (CPT), LNCaP95 cells showed stronger resistance to DNA damage than LN95(KO) and LNCaP cells, reflected in cPARP and pH2AX levels (Figure 4D). Notably, LN95(KO) cells had higher basal levels of cPARP and pH2AX than LNCaP95 and LNCaP cells, even without CPT treatment. Similar observations were also from cells treated with etoposide (Supplemental Figure 7B), validating the role of UGT2B17 in regulating DDR.

To confirm that UGT2B17 also regulates cell mitosis, we synchronized LNCaP95 and LN95(KO) cells at the G1 phase by double-blocking with thymidine, followed by release into fresh culture media. UGT2B17 depletion caused a delay in cell cycle progression at the G2/M phase but not the S phase (Supplemental Figure 8, A and B). Similarly, we synchronized LNCaP95 and LN95(KO) cells at the M phase using a double block with thymidine combined with RO3306, then released the cells into fresh media. UGT2B17 depletion resulted in delays in cell cycle progression through the M phase into the G1 phase (Supplemental Figure 8C). Together, these results demonstrate that UGT2B17 regulates gene transcription related to DDR and mitosis, and its loss leads to enhanced DDR, cell cycle arrest, and even apoptosis.

To determine whether the gene transcription regulated by UGT2B17 depends on its androgen catabolic activity, we performed RNA-Seq analysis to compare the transcriptomes regulated by UGT2B17 and UGTm in LN95(KO) cells. GSEA results indicated that both UGT2B17 proteins could globally rescue the gene transcription changes induced by UGT2B17 knockout (Figure 4E and Supplemental Data 3), including genes associated with cell mitosis, G2/M checkpoint, and DDR (Figure 4F and Supplemental Data 4). Furthermore, IF and immunoblotting assays showed that the increased DDR and cell apoptosis induced by UGT2B17 depletion could be rescued by either UGT2B17 or UGTm (Figure 4, G and H). These results could be replicated in PC3, DU145, and 22Rv1 cell models (Supplemental Figure 9), confirming that UGT2B17 has non-androgen catabolic activities by regulating gene transcription associated with mitosis and DDR.

### UGT2B17 activates Src to regulate gene transcription.

**Figure 5:**
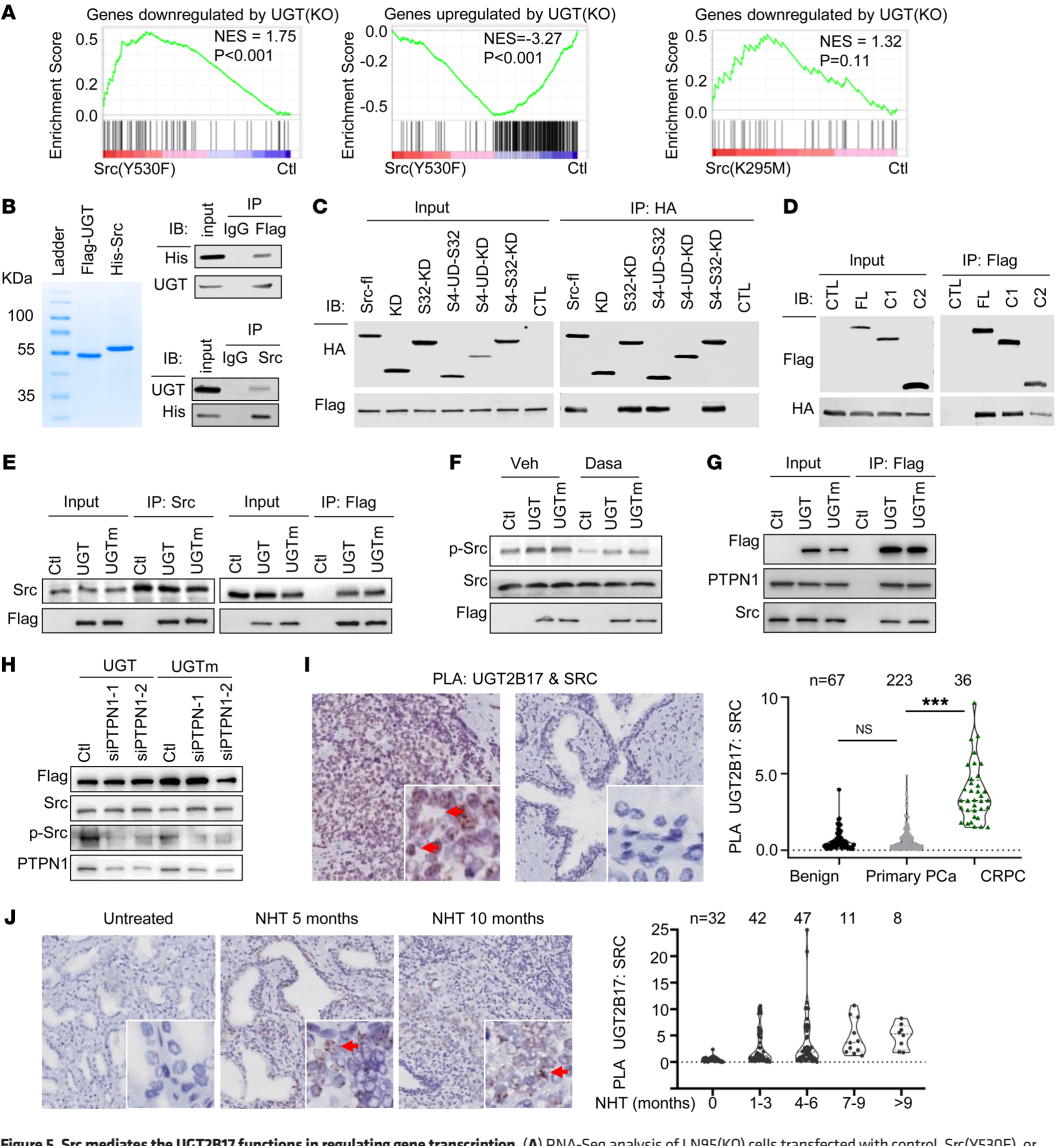
Src mediates the UGT2B17 functions in regulating gene transcription. (**A**) RNA-Seq analysis of LN95(KO) cells transfected with control, Src(Y530F), or Src(K259M) (*n* = 2 repeats). GSEA tested the association between gene sets altered by Src(Y530F) or Src(K259M) and gene sets altered by UGT2B17 knockout in LNCaP95 cells. (**B**) Coomassie staining of purified UGT2B17 and Src proteins was shown on an SDS gel. Purified Flag-UGT2B17 and His-Src were used to perform co-IP assays using Flag and His tag antibodies, respectively. (**C**) Co-IP of HA-tagged full-length and deletion mutants of Src with Flag-tag UGT2B17, followed by immunoblotting with the indicated antibodies. (**D**) Co-IP of Flag-tag full-length and deletion mutants of UGT2B17 with HA-tagged Src. Immunoblotting assays were performed with the indicated antibodies. (**E**) Co-IP of Flag-tag UGT2B17 and UGTm with Flag or Src followed by immunoblotting analysis with the indicated antibodies. (**F**) LN95(KO) cells were transfected with control, UGT2B17, or UGTm, followed by treatments of control or 10 nM of dasatinib. Immunoblotting assays were conducted using antibodies against Src, pSrc(419), and Flag. (**G**) LN95(KO) cells were transfected with control, UGT2B17, or UGTm. Co-IP of Flag-tag UGT2B17 and UGTm with PTPN1 and Src followed by immunoblotting analysis with the indicated antibodies. (**H**) LN95(KO) cells were transfected with UGT2B17 or UGTm in the presence of control or siRNA against PTPN1, followed by immunoblotting with antibodies against Flag, Src, pSrc(419), and PTPN1. (**I**) PLAs were performed on PCa tissue microarrays containing tissue cores from benign prostate, primary PCa, and CRPC samples using UGT2B17 and Src antibodies. Pathology scoring of UGT2B17-Src protein interactions was plotted. (**J**) PLAs were performed on PCa tissue microarrays containing PCa tissue cores from tumors treated with various duration of neoadjuvant hormone therapy. Pathology scoring of UGT2B17-Src protein interactions was plotted. Experiments in **B**–**H** were performed 3 times with similar results obtained. Data are shown as the mean ± SEM. Statistics performed by 1-way ANOVA test. ***P < 0.001.

UGT2B17 depletion reduced PCa cell proliferation (Figure 2) and simultaneously upregulated gene transcription related to mitosis and DDR (Figure 4), suggesting that these transcriptional changes may be compensatory responses to increased DNA damage, leading to the activation of checkpoint signaling pathways and subsequent cell cycle arrest. We hypothesized that UGT2B17 may regulate these cell checkpoints via Src, as UGT2B17 forms a protein complex with and activates Src in vitro (17). In non-PCa cells, Src has also been shown to be required for terminating the G2 checkpoint arrest in the presence of DNA damage (24). Blocking Src activity induces persistent phosphorylation and activation of ATM and ATR, which leads to subsequent cell cycle arrest. To test whether Src mediates UGT2B17’s function in regulating transcription, we conducted RNA-Seq analysis on LN95(KO) cells overexpressing either constitutively active Src (Y530F) or dominant-negative Src (K259M) (Supplemental Data 3). GSEA showed that Src (Y530F), but not Src (K259M), rescued genes that were either upregulated or downregulated by UGT2B17 depletion (Figure 5A), with the majority of these genes related to mitosis and DDR. These results suggest that Src is a key downstream effector mediating UGT2B17’s impact on gene transcription.

Src can be activated by various mechanisms, including disruption of intramolecular interactions between its SH1 domain and SH2/SH3 domains or by phosphorylation at Tyr419 or Tyr530 (25). To determine whether UGT2B17 directly interacts with and activates Src, we purified Flag-tagged UGT2B17 and His-tagged Src proteins and performed pull-down assays, confirming that UGT2B17 and Src form a direct protein complex (Figure 5B). Mapping the interaction sites between UGT2B17 and Src, we found that both the SH3 and SH2 domains of Src interacted with the cofactor domain of UGT2B17 and weakly with the substrate domain (Supplemental Figure 10A and Figure 5, C and D). We also confirmed that the UGT2B17-Src interaction is independent of the androgen catabolic activity of UGT2B17 (Figure 5E). Reduced Src activation by UGT2B17 depletion (Supplemental Figure 4A) could be rescued by overexpression of either UGT2B17 or UGTm, and these effects could be blocked by dasatinib (Figure 5F and Supplemental Figure 11). Further deletion mutagenesis studies indicated that the substrate domain of UGT2B17 is the minimal domain required to activate Src (Supplemental Figure 10B). Together, these results showed that UGT2B17 could directly interact with and activate Src and that UGT2B17’s action is independent of its androgen catabolic activity.

To investigate whether UGT2B17 may also recruit other protein kinases or phosphatases to activate Src, we found that PTPN1 was among the UGT2B17-associated proteins identified in our MS analysis (Supplemental Data 1). PTPN1 is known to dephosphorylate Src at Tyr530, thereby activating Src activity (26, 27). Using co-IP assays, we confirmed that both UGT2B17 and UGTm can form a complex with PTPN1 and Src (Figure 5G). Silencing PTPN1 by RNA interference in the presence of either UGT2B17 or UGTm reduced Src pTyr419 phosphorylation and kinase activation (Figure 5H). These changes were accompanied by increased Src phosphorylation at Tyr530 (Supplemental Figure 12). These results reveal that UGT2B17 could activate Src via possibly 2 pathways: one involving direct interaction with the SH2 and SH3 domains of Src and the other through recruitment of PTPN1. Importantly, these UGT2B17 actions are independent of its androgen catabolic activity.

To demonstrate the clinical relevance of the UGT2B17-Src complex during CRPC progression, we performed PLA using UGT2B17 and Src antibodies on patient biopsies (28). Compared with benign prostate and primary PCa tissues, the UGT2B17-Src protein complex was upregulated in CRPC (*P* < 0.001) (Figure 5I). This interaction was positively associated with the duration of androgen inhibition therapy (Figure 5J). These findings were consistent with IHC results showing upregulation of UGT2B17 protein in CRPC (Figure 1) and coincided with increased phospho-Src levels, as previously reported (17). Together, these results suggest that UGT2B17 protein levels accumulate in response to prolonged androgen inhibition therapy, which in turn triggers the UGT2B17-Src interaction and Src kinase activity.

### Blocking non-androgen catabolic function of UGT2B17 inhibits PCa cell growth and tumor progression.

**Figure 6:**
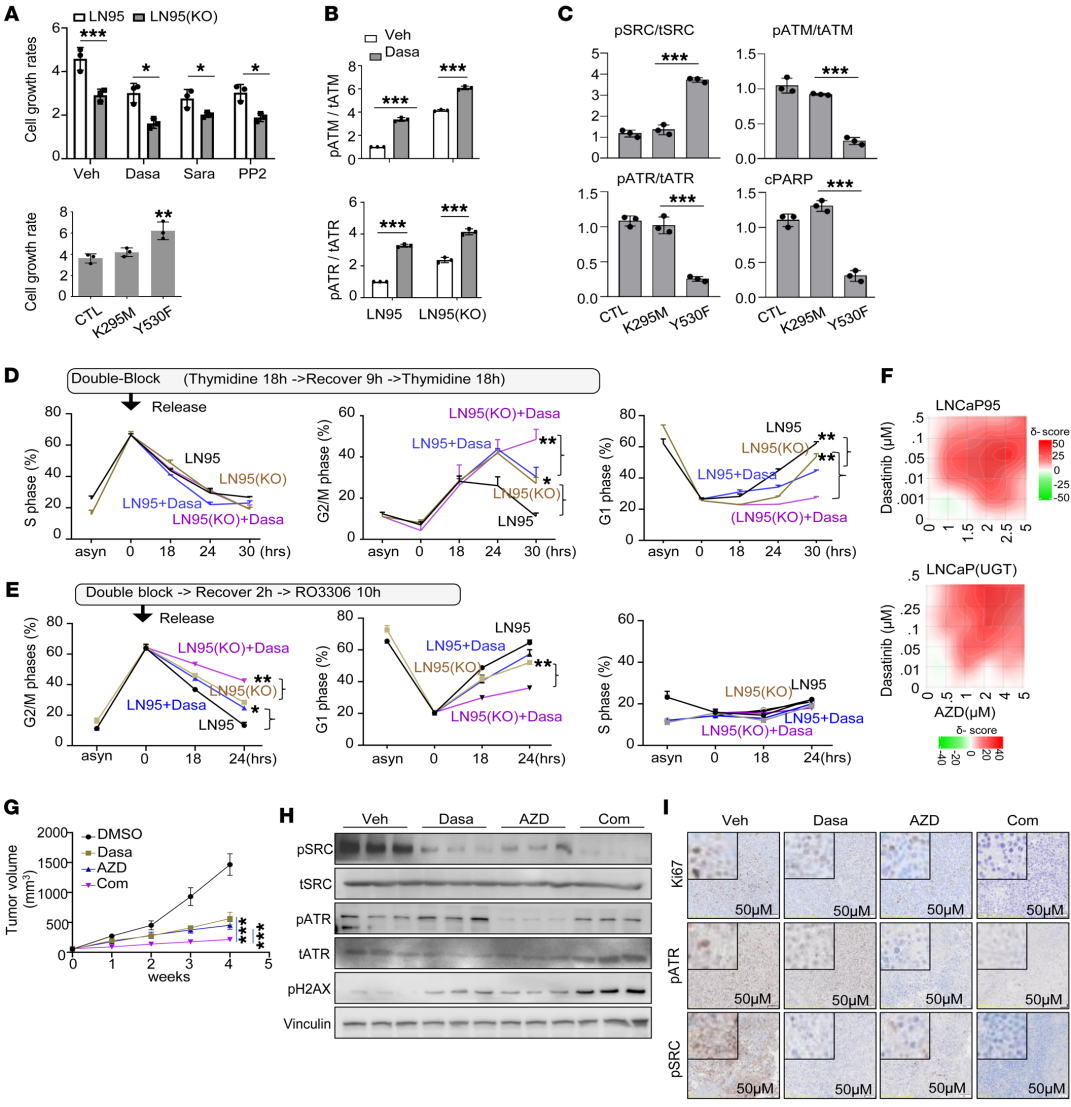
The UGT2B17/Src signaling regulates cell mitosis and CRPC xenograft growth. (**A**) LNCaP95 and LN95(KO) cells were treated with three Src inhibitors (top). LN95(KO) cells were transfected with control, constitutively active Src(Y530F), or kinase-dead Src(K259M) (bottom). Cell proliferation at day 3 was measured and normalized to that at day 0. (**B**) LNCaP95, MR49F, and their UGT2B17-knockout derivatives were treated with 10 nM dasatinib. Total and phosphorylated ATM and ATR were assessed by immunoblotting. Densitometric ratios of phospho-ATM/ATM and phospho-ATR/ATR were calculated and plotted. (**C**) LN95(KO) cells transfected with control, Src(Y530F), or Src(K259M) were analyzed by immunoblotting for Src, pSrc(Y419), ATM, pATM(S1981), ATR, pATR(T1989), and cPARP. Phosphorylation levels were quantified relative to total protein levels. (**D** and **E**) LNCaP95 and LN95(KO) cells were synchronized at the G1/S phase (**D**) or at the G2/M phase (**E**). Cells were then treated with vehicle or 10 nM dasatinib and allowed to recover for the indicated times. Cell-cycle distributions were determined by FACS. (**F**) LNCaP95 and LNCaP(UGT) cells were treated with increasing concentrations of dasatinib and AZD6738 for 3 days. Cell proliferation was measured and used to assess drug-drug interactions. (**G**) Castrated nude mice bearing LNCaP95 xenografts were treated with vehicle, dasatinib (10 mg/kg), AZD6738 (25 mg/kg), or the combination (n = 6/group). Tumor volumes were measured weekly. (**H** and **I**) After 4 weeks of treatment, tumors from each group were collected to evaluate Src, pSrc(Y419), ATR, pATR(T1989), and γH2AX (Ser139) by immunoblotting (**H**) and IHC (**I**). Experiments in **A**–**F** were repeated three times with similar results. Data are presented as mean ± SEM. Statistical analyses were performed using two-way ANOVA (**A** top, **B**, **D**, **E**, **G**) or one-way ANOVA (**A** bottom). *P < 0.05, **P < 0.01, ***P < 0.001.

Since UGT2B17 depletion regulates the transcription of genes related to cell mitosis, primarily through Src (Figure 5A), we hypothesized that Src inhibition would strongly suppress PCa cell growth. Three Src inhibitors, dasatinib, saratinib, and PP2, were shown to inhibit proliferation of LNCaP95 and MR49F cells, with effects further enhanced by UGT2B17 depletion (Figure 6A, top, and Supplemental Figure 13A). Conversely, Src (Y530F), but not Src (K259M), rescued the reduced cell proliferation of LNCaP95 cells caused by UGT2B17 depletion (Figure 6A, bottom). To confirm that the UGT2B17/Src signaling pathway controls cell cycle checkpoints through ATR and/or ATM, we challenged UGT2B17-high LNCaP95 and MR49F cells with dasatinib and observed increased levels of phospho-ATM and phospho-ATR (Figure 6B and Supplemental Figure 13B), and these changes were further amplified by UGT2B17 depletion. Consistently, Src (Y530F), but not Src (K259M), enhanced dephosphorylation of ATM and ATR in LN95(KO) cells, accompanied by reduced levels of cPARP (Figure 6C). These results indicate that the UGT2B17/Src signaling pathway dephosphorylates and suppresses ATM and ATR, facilitating cell cycling.

To determine whether Src-mediated deactivation of ATR and ATM regulates the G1/S or G2/M checkpoint in PCa cells, we synchronized LNCaP95 and LN95(KO) cell cycles at the G1 phase using a double-thymidine block, then released them. UGT2B17 depletion caused a delay in the cell cycle’s progression into the G2/M phase, but not the S phase. This delay was further strengthened by dasatinib treatment (Figure 6D), with no difference observed in the G1 phase. Similarly, when cell cycles were synchronized at the G2/M phase using a double-thymidine block combined with RO3306 and then released, UGT2B17 depletion delayed progression through the G2/M phase before entering the G1 phase. This suppression was also amplified by dasatinib (Figure 6E), indicating that the UGT2B17/Src signaling pathway regulates the G2 checkpoint, promoting PCa cell cycling.

To explore the therapeutic potential of targeting UGT2B17 signaling to suppress CRPC progression in animal models, we noted challenges in directly targeting UGT2B17 because of the lack of specific inhibitors. UGT2B17 is also highly expressed in the liver and gastrointestinal tracts. However, targeting the non-androgenic catabolic activity of UGT2B17, specifically the pathways promoting PCa progression (e.g., Src and ATR), may offer therapeutic opportunities. To test this hypothesis, we treated LNCaP95 and LNCaP(UGT) cells with increasing doses of dasatinib combined with the ATR inhibitor AZD6738 (Figure 6F). Both drugs suppressed cancer cell viability and showed synergistic effects, with synergy scores of 18.58 and 15.03, respectively, as calculated by SynergyFinder. While dasatinib monotherapy failed in phase III PCa trials (29), our results suggest that tumors with high UGT2B17/Src signaling are more likely to respond to a combination of dasatinib and AZD6738 treatments. Notably, AZD6738 is currently being tested in combination therapies with other anticancer agents in various cancers. Using LNCaP95 xenograft models, we confirmed tumor growth inhibition by dasatinib or AZD6738 alone, with even greater suppression when these 2 drugs were combined (Figure 6G). Immunoblotting and IHC analyses confirmed that tumor suppression was associated with reduced Src and ATR activities in the tumors (Figure 6, H and I). These results highlight that targeting UGT2B17 downstream signaling by inhibiting Src and ATR has strong tumor suppression effects, suggesting a potential therapeutic strategy for CRPC.

## Discussion

To develop therapy resistance, cancer cells commonly employ “counteraction” or “adaptation” strategies. This is exemplified by prostate tumors in response to androgen inhibition therapy. These tumors may enhance androgen production and AR protein synthesis to strengthen androgen/AR signaling, counteracting AR inhibitors. Alternatively, they may adapt by activating AR in an androgen-independent manner. In more extreme cases, tumor cells switch off AR expression entirely and transition into more aggressive, AR-independent neuroendocrine prostate tumors. However, overcoming the addiction to androgens can be lethal for androgen-dependent PCa cells, as evidenced by androgen inhibition therapy, which initially suppresses tumors effectively. However, the inevitability of CRPC indicates that some tumor cells possess intrinsic adaptation mechanisms that enable them to survive and thrive in the absence of androgens.

In this study, we demonstrate a dual role of UGT2B17 under androgen inhibition therapy. Through its androgen catabolic activity, UGT2B17 accelerates androgen clearance inside PCa cells, reducing their reliance on androgens. Simultaneously, it exerts non-androgen catabolic functions that activate tumor-promoting pathways, facilitating CRPC progression. Thus, this study highlights an adaptation strategy employed by PCa cells to develop therapy resistance.

UGT2B17 exerts these non-androgen catabolic activities through interactions with various functional complexes (e.g., PDIs and Src). This flexibility may be attributed to the presence of multiple UGT2B17 protein conformations coexisting in PCa cells, allowing for diverse subcellular localizations and interacting protein partners. Evidence for this is provided by 2 highly specific UGT2B17 antibodies (11–13), which detect differential subcellular localizations of the UGT2B17 protein in PCa cells (Figure 1). According to DisEMBL (https://mybiosoftware.com/disembl-1-5-protein-disorder-prediction.html), UGT2B17 possesses at least 3 intrinsic protein disorder (IPD) regions at aa68–85, aa180–196, and aa263–291. IPD regions do not adopt a fixed 3D structure and exist as a dynamic ensemble of protein conformations that permit flexible protein-protein interactions (30). Additionally, the C-tail domain of UGT2B17 contains an IPD region at aa519–530, which overlaps with both an NLS and an ER retention peptide, enabling UGT2B17 to shuttle between different subcellular compartments to exert both androgen and non-androgen catabolic activities.

Our study provides several important insights on therapies against CRPC. It will aid in the development of biomarkers for patient stratification for bipolar androgen therapy (BAT) and rationalize the combination of BAT with other agents to enhance therapy efficacy. BAT involves cyclic administration of testosterone cypionate and luteinizing hormone-releasing hormone (LHRH). agonists every 28 days to induce rapid fluctuations in serum T levels from supraphysiological (>1,500 ng/dL) to near-castration levels (31). Clinical trials have shown that BAT results in a prostate-specific antigen response in only 20%–25% of patients (32–34). Our results suggest that tumors with high UGT2B17 expression should be excluded from BAT trials, as these tumors efficiently eliminate androgens, leading to reduced oscillation of androgen concentrations inside PCa cells. While BAT manipulates AR activity by altering androgen levels, UGT2B17 renders PCa cells insensitive to androgens. Supraphysiological T enhances double-strand DNA breaks through TOP2B, supporting the rationale for combining BAT with etoposide or olaparib. However, tumors with high UGT2B17 expression are resistant to DNA damage-induced cell cycle arrest. In contrast, these tumors may be more sensitive to dasatinib in addition to BAT.

This study also provides insights into developing combination therapies involving dasatinib. Although dasatinib failed in phase III trials as a monotherapy for chemotherapy-naive men with metastatic CRPC, it has been approved by the FDA for chronic myeloid leukemia and acute lymphoblastic leukemia and has demonstrated acceptable side effects and promising therapeutic potential in phase I and II PCa trials (35, 36). These results underscore the heterogeneous nature of metastatic PCa and the need for biomarkers to guide patient selection for Src inhibition therapy. While UGT2B17-high tumors exhibit constitutively active Src that may be more sensitive to dasatinib, the UGT2B17-Src protein complex detectable by using PLA could serve as a biomarker for selecting patients who may benefit from dasatinib treatment. Moreover, our findings suggest that combined Src and ATR inhibition may result in more comprehensive tumor suppression.

Collectively, this study identifies several non-androgen catabolic functions of UGT2B17 that promote CRPC progression. These findings provide valuable insights for the development of future therapies and diagnostic strategies for PCa.

## Methods

### Sex as a biological variable.

Our study exclusively examined male mice because prostate cancer is relevant only in male humans.

### Tissue microarray and IHC.

Prostate tumor samples were retrieved from the Vancouver Prostate Centre (VPC) tissue bank and used to construct several tissue microarrays (TMAs) as previously described (17, 37–39). Detailed clinical information for each patient with PCa was included in these studies. CRPC tissue cores were collected from patients who had undergone hormonal therapies and were diagnosed with CRPC. The recurrent tumors were removed by transurethral resection prostatectomy to alleviate obstructive symptoms. The treatment-naive TMA included tissue cores from patients who had undergone radical prostatectomy. Neoadjuvant hormone therapy (NHT) tissue cores were collected from 87 patients who received 0–12 months of NHT treatment. TMA slides were stained using a Ventana Discovery XT autostainer, as previously reported (17, 37–39). The UGT2B17 antibodies (EL95 and EL-2B17mAb) were provided in-house, and their specificity for prostate cancer tissues had been previously validated (9, 15, 17, 40). All stained slides were scanned with a Leica SCN400 scanner, and digital images were evaluated by a pathologist. The IHC signal for UGT2B17 was scored using Aperio ImageScope software, based on the intensity and percentage of IHC signals according to the manufacturer’s instructions (Leica Biosystems), as reported previously (37, 41, 42).

### Cell lines and chemicals.

Human PCa cell line LNCaP, DU145, PC3, and 22Rv1 were purchased from the American Type Culture Collection (ATCC) and maintained in RPMI-1640 medium supplemented with 10% fetal bovine serum (FBS). LNCaP95 and MR49F cells were provided by Drs. Alan Meeker (Johns Hopkins University, Baltimore, Maryland, USA) and Martin Gleave (University of British Columbia [UBC], Vancouver, British Columbia, Canada), respectively. LNCaP95 cells were cultured in RPMI-1640 medium supplemented with 5% charcoal-stripped serum (Hyclone), while MR49F cells were maintained in medium containing 10 μM enzalutamide. HEK293T cells were obtained from ATCC and cultured in DMEM with 10% FBS. LNCaP(UGT2B17) cells was constructed by our lab and reported before (17). All cell lines were cultured at 37°C in a humidified incubator with 5% CO_2_ and routinely authenticated by short tandem repeat assays. The chemicals used in this study are listed in Supplemental Table 1.

### Plasmid vector and siRNA.

UGT2B17 cDNA was used as a template to construct deletion mutations, which were then subcloned into pEGFP and pCMV1 vectors and validated by Sanger DNA sequencing. Expression vectors for wild-type and mutant UGT2B17 and Src were purchased from Addgene or LST Bio-tech Shandong Co., Ltd., and validated by immunoblotting. CRISPR vectors targeting UGT2B17 were provided by Nima Shariff (Cleveland Clinic, Cleveland, Ohio, USA) and have been previously reported (43). Human IRE1a-GFP constructs and PTPN1 siRNA (sequence: 5′-NNUGACCAUAGUCGGAUUAAA-3′) were synthesized by LST Bio-tech Shandong Co., Ltd. Lipofectamine 3000 (Thermo Fisher Scientific) was used for siRNA and plasmid transfections, following the manufacturer’s protocol. Media were refreshed after 6–8 hours, and cell samples were collected at 36 hours for real-time PCR or 72 hours for Western blotting/co-IP following transfection.

### IF.

LNCaP95 cells were seeded onto coverslips in 24-well plates and cultured for 24 hours. The cells were then fixed with 4% paraformaldehyde for 15 minutes and permeabilized with PBS containing 0.25% Triton X-100 for 10 minutes at room temperature. After 3 rinses with PBS, the cells were blocked with 1% BSA for 1 hour at room temperature. Primary antibodies were incubated overnight at 4°C. The following day, the slides were rinsed with PBS and incubated with Alexa Fluor 568–conjugated goat anti-rabbit antibody (1:500 in PBS containing 1% BSA; Abcam) for 40 minutes at 37°C. After 3 washes with PBS, coverslips were mounted with mounting medium containing DAPI (Vector Laboratories, H-1200-10). Cell images were captured using a digital lens (Carl Zeiss).

### Western blotting.

Cells were collected using lysis buffer (50 mM Tris pH 8.0, 150 mM NaCl, 1% NP-40, 0.1% SDS) supplemented with 1% protease inhibitor cocktail. Protein concentrations were determined using a BCA kit (Bio-Rad). Twenty to sixty micrograms of protein from the cell lysate were mixed with 4× SDS loading buffer (200 mM Tris, 5.7 mM bromophenol blue, 40% glycerol, 10% SDS, 8 mL β-mercaptoethanol) and heated at 95°C for 5 minutes. Protein samples were subjected to SDS-PAGE, and the gels were transferred to PVDF membranes (Millipore). After transfer, the membranes were blocked with 5% nonfat milk (Bio-Rad) in Tris-buffered saline with 0.1% Tween 20 (TBST) for 1 hour at room temperature. Membranes were then incubated with the indicated primary antibodies overnight at 4°C. The following day, the membranes were washed with 1× TBST 3 times for 10 minutes each and incubated with goat anti-mouse/rabbit IgG (H+L)-HRP secondary antibody (Abcam, 1:5,000 dilution) for 1 hour at room temperature. After washing with 1× TBST 3 times for 10 minutes, the signals were developed using ECL substrate (Millipore) and captured using a Bio-Rad imaging system. Antibody details are provided in Supplemental Table 2.

### Co-IP assay.

Cells were lysed in NETN buffer (1% NP-40, 1 mM of EDTA, 50 mM of Tris, and 150 mM of NaCl plus proteinase and phosphatase inhibitor). After sonication and centrifugation to collect supernatant, we added 4 μg control IgG (mouse or rabbit normal IgG depends on the species of IP antibody) and 50 μL Sepharose A/G beads rotating for 2 hours at room temperature. Next day, the indicated antibodies were mixed into cell lysates and incubated at 4°C for overnight. Then, we centrifuged at 1,000*g* for 1 minute to discard the supernatant and washed the lysates mixed with NETN buffer 3 times. To denature proteins, beads were mixed with 1× SDS loading buffer and heated at 95°C for 5 minutes. Protein samples were subjected to Western blot for further analysis.

### Real-time PCR.

Total RNA was extracted from cells using the Purelink RNA mini kit (Invitrogen) and reverse-transcribed into cDNA using ReverTra Ace qPCR RT Kit (Toyobo, catalog FSQ-101). The SYBR Green Mix (Roche) and Biosystems 7900HT (Thermo Fisher Scientific) were utilized to conduct the real-time PCR following manufacturer’s instruction. The housekeeping gene GAPDH was used as a control, and the relative RNA level was calculated using the 2^-ΔΔCt^ method with the Ct values normalized to GAPDH. All real-time PCR assays were carried out using 3 technical replicates and 3 independent cDNA syntheses. Primer sequences are provided in Supplemental Table 3.

### Proliferation assay.

Incucyte Live-cell Imaging system (Essen Bioscience) was used to measure cellular proliferation rates of PCa cells. Cells were seeded on 96-well plates and treated with compounds as indicated. The plates were incubated in culture incubators for indicated days. During the incubation period, the cells were photographed, and cell density was analyzed by using Incucyte 2011A software. Cell confluence was calculated and expressed as an increase in percentage of confluence as compared with control.

### In vivo ubiquitination assay.

In vivo ubiquitination assays were carried out as we reported previously (44, 45). Briefly, LNCaP cells stably expressing Flag-tagged UGT2B17 were cultured in media containing 10% charcoal-stripped serum for 0 or 28 days. Cells were transfected with plasmids encoding HA-tagged ubiquitin and treated with 2 μg/mL of MG132 for 16 hours. Whole-cell lysates were extracted using a buffer containing 50 mM of Tris pH 8.0, 150 mM of NaCl, 1% NP-40, 0.5% sodium deoxycholate, 2% SDS, and proteinase and phosphatase inhibitors (Roche). Lysates were diluted 10 times with a buffer containing 50 mM of Tris pH 8.0, 150 mM of NaCl, and 1% NP-40 and subjected to IP with Flag antibody. The associated proteins were then immunoblotted with UGT2B17 antibody.

### Cell synchronization and flow cytometry.

A double-thymidine block was used to arrest cells at G1/S transition. LN95 and LN95-UGT KO cells were seeded on 6-well plates and cultured overnight. Cells were treated with 2 mM thymidine for 16 h; then we removed thymidine by washing cells with PBS. Subsequently, fresh medium was added for 9 h, then treated with 2 mM thymidine for another 16 h. Cells were washed with PBS followed by the addition of regular culture media to release cell cycling. Cells were collected at indicated time for analysis of cell cycle by DNA staining using propidium iodide (PI).

To arrest the cell cycle at the G2/M border, cells were treated with a double-thymidine block, released into fresh medium for 4 hours, and then treated with RO-3306 (10 nM) for 10 hours. Cells were washed with PBS followed by the addition of regular culture media to release cell cycling. Cells were collected at indicated times for analysis of cell cycle by DNA staining using PI. At indicated time points after treatment, the cells were harvested using trypsin and washed twice with PBS. The cells were then suspended and fixed with 70% ethanol in PBS at 4°C overnight. The next day, the cells were centrifuged 2,500*g* for 5 minutes and the supernatant was removed, and then we added 1 mL buffer solution (0.1% Triton X-100, 0.1% sodium citrate in PBS buffer) that contained 25.0 μg/mL RNase A and 40.0 μg/mL PI. Relative DNA contents were analyzed by FACSCanto II flow cytometer and BD FACSDiva software v5.0.3.

### RNA-Seq data analysis.

RNA-Seq reads were mapped to reference genome hg19 with hisat2 (version 2.1.0) (46) using default settings. The mapped transcriptome was then counted with htseq (version 0.11.2.) and gencode v29 gtf (47). Using the raw count matrixes, we identified differentially expressed genes with DESeq2 (version 1.24.0) (48). Volcano plots were generated from DESeq2 Log2FoldChange values and –log10 *P*-adjusted values with R’s ggplot2 package (version 3.2.0). GSEA was conducted using GSEA version (4.1.0) with RNK file input against the Hallmark gene sets (49). RNK files were generated from Log2FoldChange values and sorted with descending order (49). Gene count data were scaled to sample total read counts with DESeq2 count function and then *z*-normalized between samples.

### Liquid chromatography–MS analysis.

On-bead trypsin digestion was carried out in 50 μL HEPES pH 8 with 1 μg trypsin at 37°C overnight. Digested peptide concentration was determined using a NanoDrop/BCA assay, put through 200 μL C18 TopTips, and eluted peptides were dried (Centrivap) and dissolved at 0.7 μg/μL with 0.1% formic acid. Analysis of peptides was carried out on an Orbitrap Fusion Lumos MS platform (Thermo Fisher Scientific) coupled to an Easy-nLC 1200 system (Thermo Fisher Scientific) using an in-house 100 μm ID × 100 cm column (Dr. Maisch 1.9μ C18) and EasySpray source (Thermo Fisher Scientific). The analytical column was equilibrated at 700 bar for a total volume of 8 μL, and the injection volume was 2 μL. A gradient of mobile phase A (water and 0.1% formic acid) and B (80% acetonitrile with 0.1% formic acid) at 0.25 μL/min, 2%–27% B from 2–90 minutes followed by 27%–40% B over 12 minutes, 40%–95% B over 8 minutes, and 10 minutes at 95% was used. Data acquisition was carried out using a data-dependent method with MS2 in the Orbitrap using a positive-ion spray voltage of 2,100, transfer tube temperature of 325°C, and default charge state 2. Survey scans (MS1) were acquired at a resolution of 120,000 across a mass range of 350–1,500 *m/z*, with RF 30, an AGC target of 4e5, and a maximum injection time of 50 ms in profile mode. For MS2 scans there was an intensity threshold of 5e4; charge state filtering of 2–5; dynamic exclusion 30 seconds with 10 ppm tolerances with a 1.2 *m/z* window prior to stepped HCD fragmentation of 31%, 33%, 37% using 7,500 resolution; auto mass range; AGC target 5e4; and maximum injection time of 124 ms in centroid mode. All data files were processed with Protein Discoverer 2.5. Spectrum files were recalibrated and features extracted with Minora. Searches were carried out with Sequest HT with SwissProt TaxID=9606 (v2017-10-25) with precursor mass tolerance 10 ppm and fragment mass tolerance 0.01 Da, carbamidomethyl static modification, and K,M,P oxidation and S,T,Y Phos dynamic modification. Decoy database strict and relaxed FDR targets were 0.01 and 0.05 based on *q* value. Precursor quantification was intensity based with unique and razor peptides used, normalizing on total peptide amount with scaling on all averages.

### PLAs.

PLAs were conducted using the Duolink PLA Brightfield kit (Sigma) according to the manufacturer’s protocol as we reported (28). Baked and deparaffinized TMA slides were first incubated in cell conditioning 1 (Ventana) solution for 64 minutes at 91°C for antigen retrieval. TMA cores were then incubated for 2 hours at room temperature in a solution of antibodies specific to targets diluted in 1× TBS: UGT2B17 (dilution: 1:50, custom made) and c-Src (dilution: 1:25, sc-8056, Santa Cruz Biotechnology). Mouse and rabbit PLA probes diluted in Duolink Antibody Diluent were incubated for 1 hour at 37°C. Probe ligation was performed for 32 minutes at 37°C using the Duolink ligation solution. PLA signals were amplified for 2 hours at 37°C with Duolink amplification solution, detected with HRP-conjugated oligonucleotides for 1 hour at room temperature using Duolink Detection Brightfield reagents, and developed with Duolink substrate reagents for 8 minutes at room temperature. Slides were counterstained in hematoxylin, washed in soapy water, and rinsed in tap water. Dried slides were mounted and then scanned. For analysis, PLA signals were scored by a pathologist using Aperio ImageScope.

### PDI assays.

PDI activity was measured by 2 commercial kits. To measure intrinsic PDI activity from PCa cells, Abcam (ab273337) was used. LN95(KO) cells were transfected with control, UGT2B17, or UGTm for 72 hours. Cell lysates were collected with ice-cold PDI Assay Buffer. A reaction containing 50 μL protein sample and 25 μL reaction mix were mixed and added to 96-well plates. After incubation at room temperature for 15 minutes, 25 μL detection mix was added to each well, and the fluorescence was detected with excitation/emission (Ex/Em) = 490/580 nm in kinetic mode. To measure purified PDI protein activity, we used the ENZO kit (ENZ-51024). Flag-tagged UGT2B17 and UGTm were purified by anti-Flag Affinity Gel (catalog HY-K0217, MCE). The PDI enzymatic activity was detected following manufacturer’s instructions. Briefly, 50 μL of diluted insulin solution was added to a 96-well plate. It was followed by adding 10 μL of the working solution of the PDI, 10 μL test agent, and 10 μL DTT. Subsequently, the microplate was incubated in the dark at room temperature for 15 minutes, and the fluorescence was detected with Ex/Em = 500/603 nm using SpectraMax iD3.

### Xenograft experiments.

Male nude mice were purchased from Beijing Vital River Laboratory Animal Technology Co. Ltd. Five million LNCaP95 cells were suspended in 200 μL of PBS-diluted Matrigel (1:1) and injected subcutaneously into the dorsal flank of castrated male nude mice aged 6–8 weeks. Tumor-bearing mice were randomly divided into 4 groups (*n* = 6 per group) and treated via i.p. injection every other day for 4 weeks as follows: (i) DMSO, (ii) AZD6738 (25 mg/kg), (iii) dasatinib (10 mg/kg), and (iv) AZD6738 (25 mg/kg) plus dasatinib (10 mg/kg). Tumor volumes were measured weekly using a digital caliper and calculated with the formula: volume = 0.5 × length × width × width. After 28 days of treatment, mice were euthanized, and tumors were collected for immunoblotting and IHC analysis.

### Statistics.

Statistical analyses were carried out using GraphPad Prism (version 8) software. Experimental data were normalized to internal controls from at least 3 independent biological replicates, with all data represented as means ± SD. When comparing data from 2 treatment groups (e.g., LNCaP95 vs. LN95(KO) cells), Student’s 2-tailed *t* test was used to determine significance when *P* < 0.05. For comparisons involving more than 2 groups, ANOVA followed by Tukey’s test for pairwise comparisons was performed.

### Study approval.

The research protocol for the VPC cohort was approved by the Clinical Research Ethics Board of the UBC (H20-01680), and all patients signed a formal consent form approved by the ethics board. All animal studies were approved by The First Affiliated Hospital of Shandong First Medical University (2024-S6056).

### Data availability.

The RNA-Seq data from this study are deposited in the Gene Expression Omnibus under accession codes GSE292707 and GSE292813. Supporting data relevant to the main manuscript and supplement are available in the Supporting Data Values file.

## Author contributions

XD performed conceptualization. TF, NX, LG, QJ, and SHYK performed data curation. LW, TM, LF, LL, and YL were responsible for methodology and resources. CG, EL, NAL, BH, and XD were responsible for supervision. TF, EL, NAL, JQ, and XD were responsible for writing and revision.

## Funding support

No funding suupport.

## Supplementary Material

Supplemental data

Supplemental data 1

Supplemental data 2

Supplemental data 3

Supplemental data 4

Unedited blot and gel images

Supporting data values

## Version 1 12/04/2025

In-Press Preview

## Version 2 01/16/2026

Electronic publication
