# Supplementary material for: Noncanonical functions of UGT2B17 promote castration-resistant prostate cancer progression
Source: J Clin Invest. 2025 Dec 4;136(2):e196495. doi: 10.1172/JCI196495 (PMC12807465; doi:10.1172/JCI196495)
Supplement: Supplemental data [file jci-136-196495-s115.pdf]

Supplementary materials

Non-canonical functions of UGT2B17 promote castrate-resistant prostate cancer progression

Tingting Feng, Ning Xie, Lin Gao, Qiongqiong Jia, Sonia Kung, Tunc Morova, Fan Zhang, Yinan Li, Lin Wang, Ladan Fazli, Eric Lévesque, Nathan Lack, Jianfei Qi, Bo Han, Xuesen Dong

CONTENTS

Figure S1-13

Table S5-7

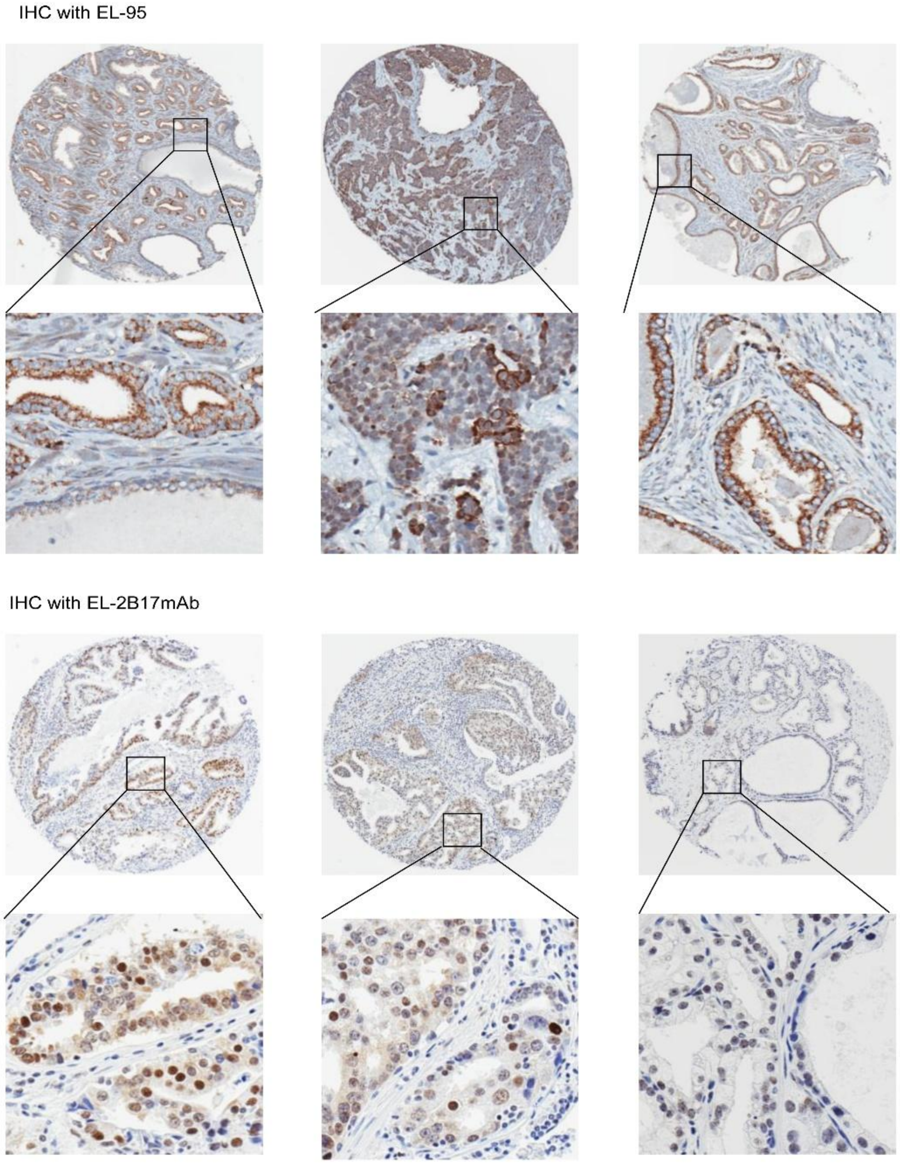

Supplementary Figure 1. UGT2B17 protein expression detected by immunohistochemistry in prostate cancer patient tumors

PCa tissue microarrays were used to detect UGT2B17 protein expression by using two UGT2B17 specific antibodies, EL-95 and EL-2B17mAb. Both low and high resolutions of IHC images were presented. High resolution images were also presented in Figure 1.

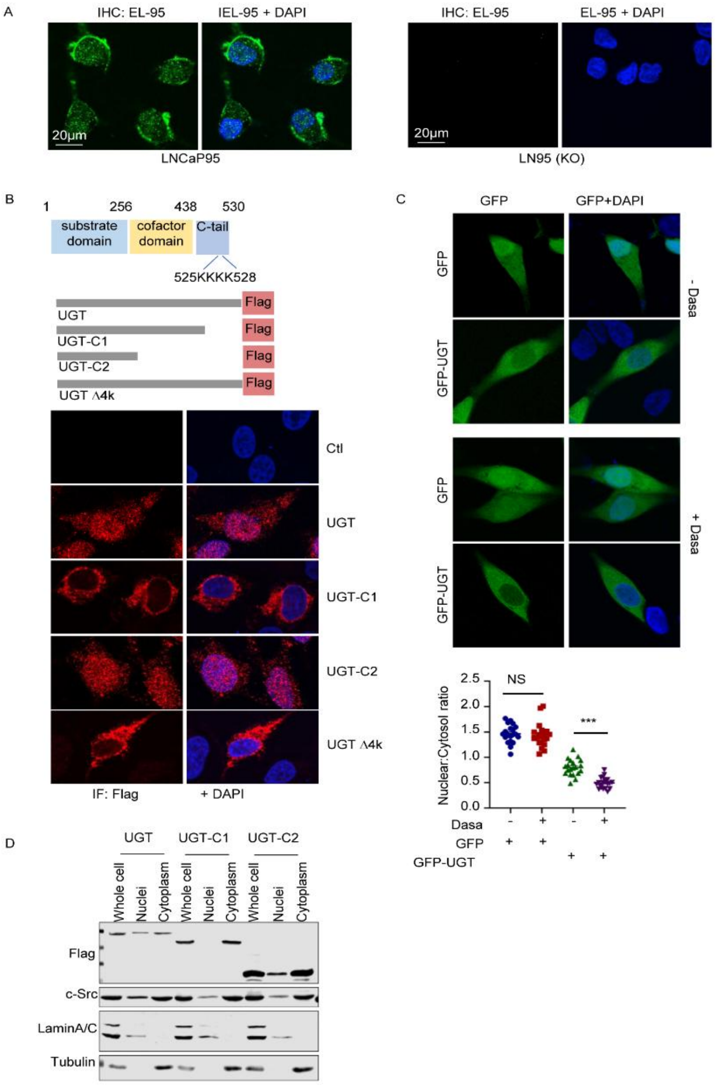

Supplementary Figure 2. Subcellular locations of UGT2B17 in prostate cancer cells

(A) Cellular localizations of UGT2B17 protein in LNCaP95 and LN95(KO) cells were detected by immunofluorescence using the EL-95 antibody. **(B)** Vectors expressing C terminal Flag-tagged UGT2B17 (aa1-530) and mutant UGT2B17 (C1, aa1-438; C2, aa1- 256; Δ4, deletion of aa525-528) were transfected in LNCaP cells for 72h. The cells were then stained with the Flag (red) antibody and counterstained with DAPI prior to analysis by confocal microscopy. **(C)** LNCaP cells were transfected with vectors expressing either GFP or GFP-tagged UGT2B17, and subsequently treated with either vehicle or 10 nM dasatinib for 1 hour. The subcellular localization of UGT2B17 proteins was examined using confocal microscopy. GFP signals in the cytosol and nuclei were quantified using ImageJ software, analyzing twenty randomly selected cells from each slide. **(D)** LNCaP cells were transfected with plasmid vectors expressing Flag-tagged UGT2B17 (aa1-530) and its mutants (C1, aa1-438; C2, aa1-256). Whole-cell lysates, along with cytoplasmic and nuclear fractions, were collected and subjected to immunoblotting with indicated antibodies. Data are shown as the mean ± SEM. Statistical tests performed by one-way ANOVA test. *** *P* < 0.001, NS: no significance.

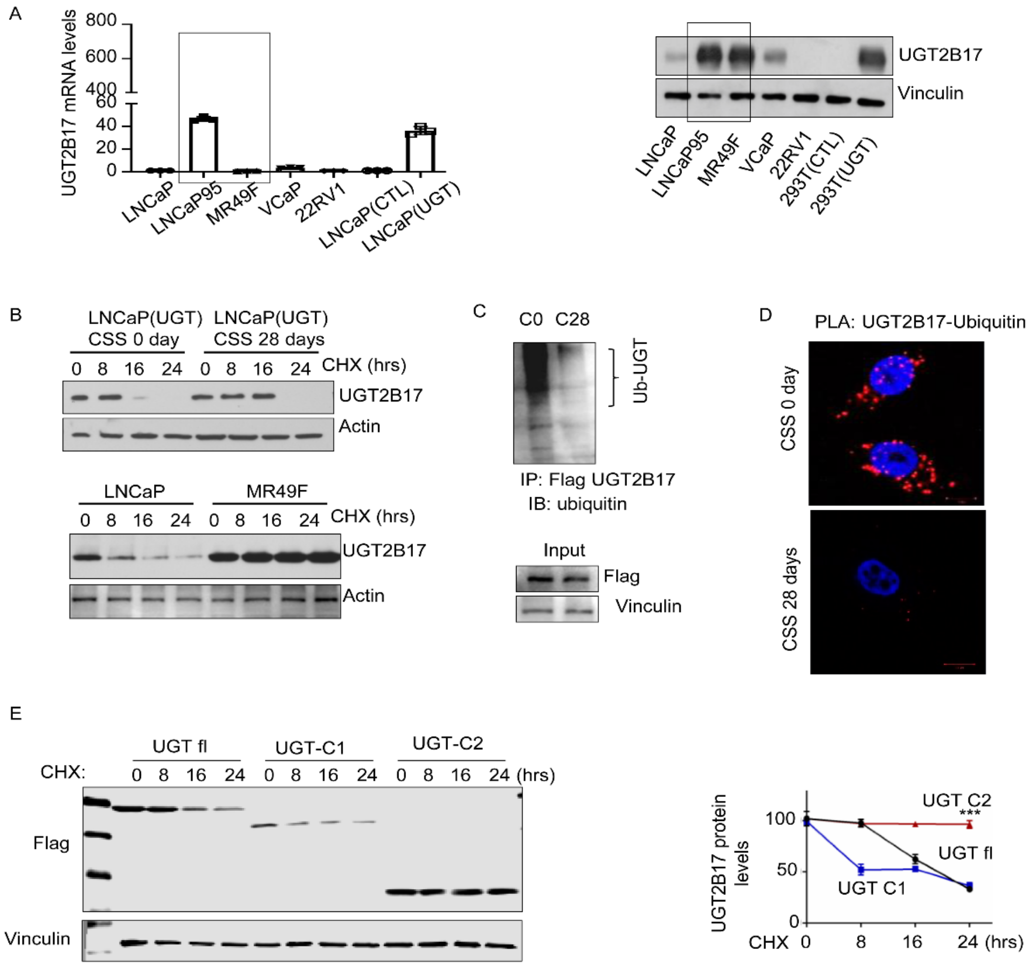

Supplementary Figure 3. Prolonged androgen depletion stabilizes UGT2B17 protein
(A) Total RNA and protein lysates were extracted from various PCa cell lines as indicated. UGT2B17 mRNA and protein levels were detected by real-time qPCR and immunoblotting. 293T cells transfected with either control or UGT2B17 expression vectors served as controls. **(B)** LNCaP (UGT2B17) cells under androgen depletion for 0 or 28 days, along with LNCaP and MR49F cells, were treated with cycloheximide for 0 to 24 hours. UGT2B17 protein levels were measured by immunoblotting with actin or vinculin as a loading control. **(C)** LNCaP cells were treated with androgen depletion for 0 or 28 days and subsequently transfected with Flag-tagged UGT2B17. In vitro ubiquitination assays were performed by co-IP using a Flag antibody, followed by immunoblotting with a ubiquitin antibody. **(D)** LNCaP cells treated with androgen depletion for 0 or 28 days were used for PLA with UGT2B17 and ubiquitin antibodies. **(E)** Plasmid vectors encoding Flag-tagged UGT2B17 (aa1-530) and its mutants (C1, aa1-438; C2, aa1-256) were transfected into LNCaP cells. Cells were treated with cycloheximide for 0 to 24 hours. Cell lysates were collected and immunoblotted with a Flag antibody, with vinculin serving as a control. Data are shown as the mean ± SEM. Statistical tests performed by one-way ANOVA test. *** $P < 0.001$.

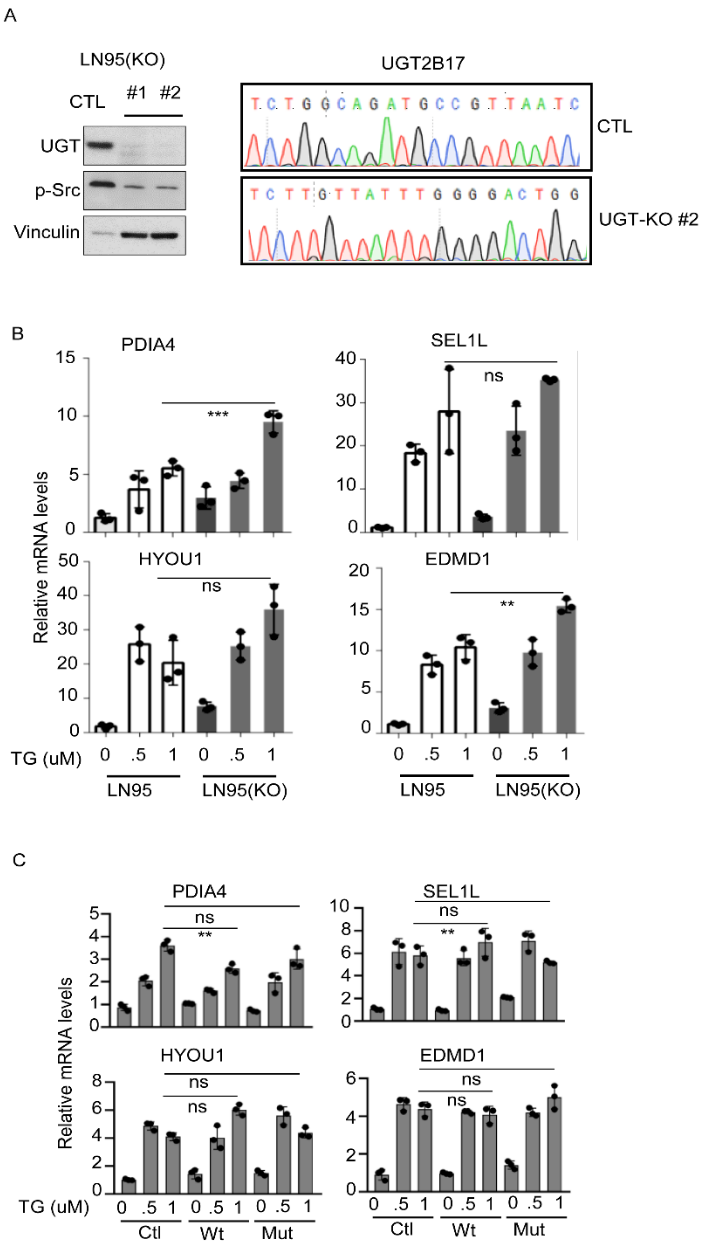

Supplementary Figure 4. UGT2B17 regulates UPR in prostate cancer cells

(A) LNCaP95 cells were used to generate UGT2B17 knockout cell lines (LN95(KO)), which were validated by immunoblotting and Sanger sequencing. **(B)** LNCaP95 and LN95(KO) cells were treated with 0-1 μM thapsigargin (TG) for 24 hours. PDIA4, SEL1L, HYOU1, and EDME1 mRNA levels were measured by real-time PCR. **(C)** LN95(KO) cells were transfected with plasmids encoding control, UGT2B17, and UGTm. Cells were then challenged with increasing doses of TG. PDIA4, SEL1L, HYOU1, and EDME1 mRNA levels were measured by real-time qPCR. Data are shown as the mean ± SEM. Statistical tests performed by two-way ANOVA test. ***P* < 0.01, ****P* < 0.001, NS: no significance.

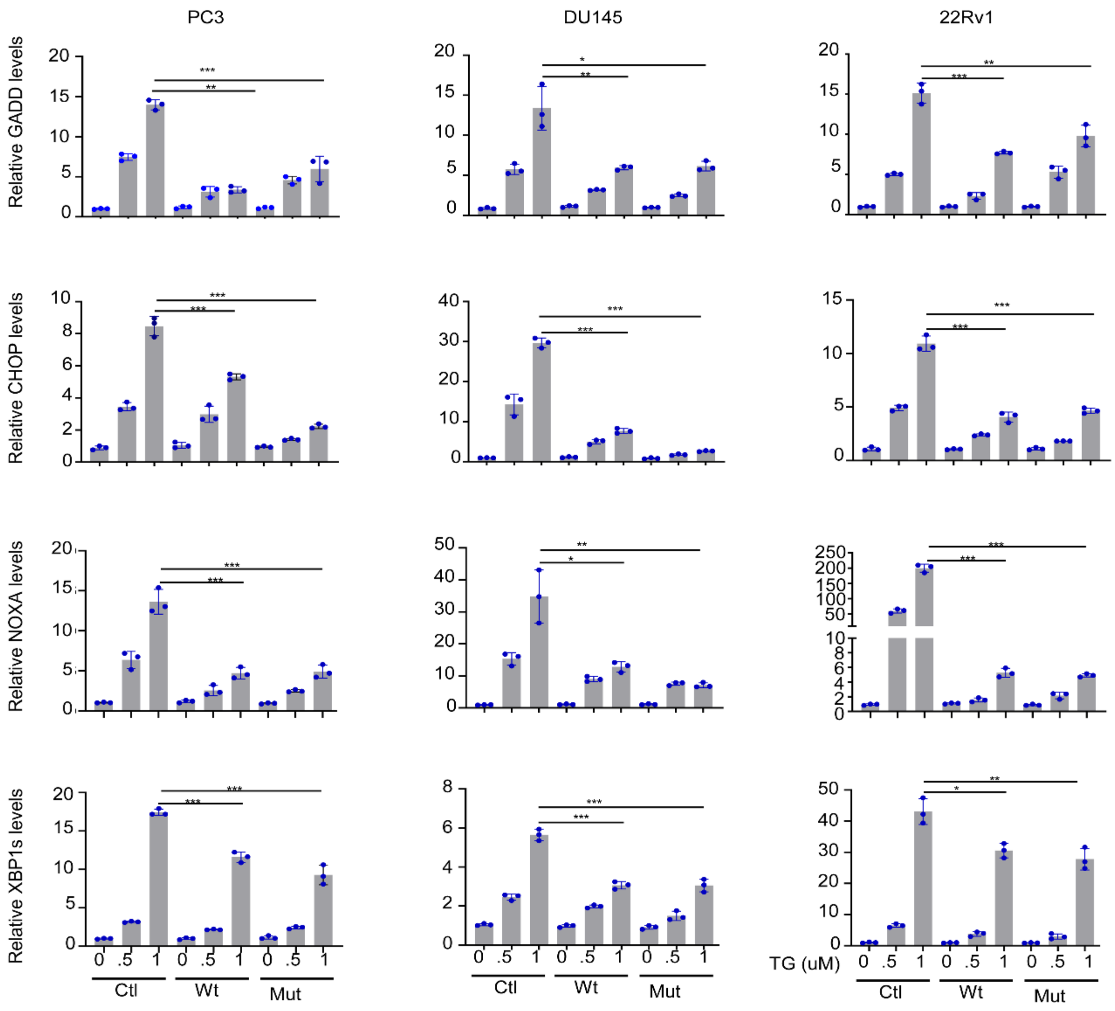

Supplementary Figure 5. UGT2B17 regulates UPR in PCa cells

PC3, DU145 and 22Rv1 cells were transfected with control, UGT2B17 and UGT2B17m for 36 hours. Cells were then treated with increasing doses of TG for 8 hours. Real-time qPCR measured the mRNA levels of UPR related genes. Data are shown as the mean ± SEM. Statistical tests performed by two-way ANOVA test. **P* < 0.05, ** *P* < 0.01, *** *P* < 0.001.

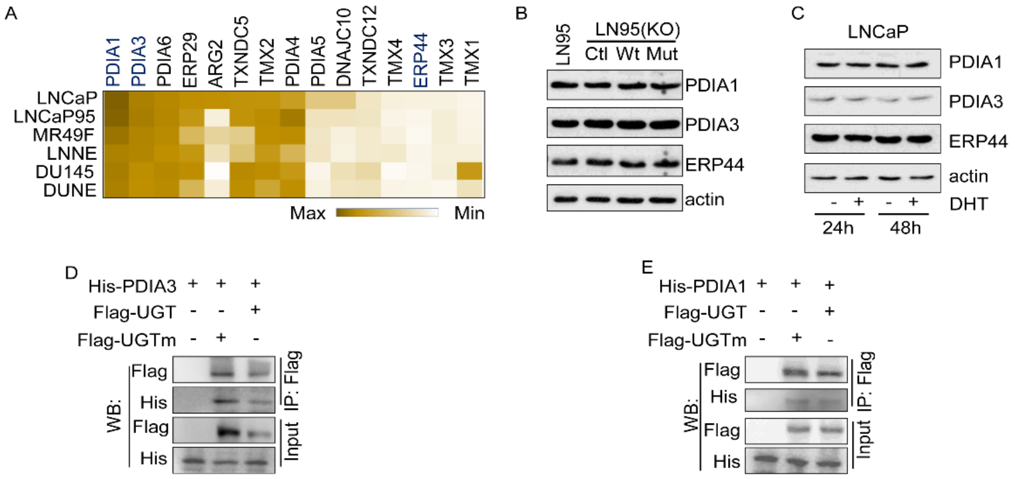

Supplementary Figure 6. The expression and protein interactions of PDIs in prostate cancer cells
(A) A heatmap displayed RNA levels of PDI family members in PCa cell lines. **(B)** LNCaP95 and LN95(KO) cells were transfected with plasmids encoding control, UGT2B17, and UGTm. PDIA1, PDIA3, and ERP44 protein levels were measured by immunoblotting. **(C)** LNCaP cells were cultured under androgen-depleted conditions for 2 days before being treated with either vehicle or 10 nM DHT for 24 or 48 hours. PDIA1, PDIA3, and ERP44 protein levels were measured by immunoblotting. **(D)** Protein pulldown assays using purified His-tagged PDIA3 incubated with either purified Flag-tagged UGT2B17 or UGTm from 293T cells. Protein complexes were precipitated by the Flag antibody and immunoblotted with Flag and His tag antibodies. **(E).** Protein pulldown assays using purified His-tagged PDIA1 incubated with either purified Flag-tagged UGT2B17 or UGTm from 293T cells. Protein complexes were precipitated by the Flag antibody and immunoblotted with Flag and His tag antibodies.

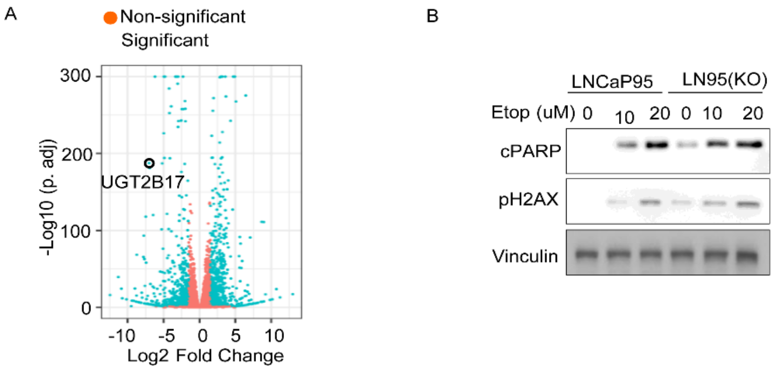

Supplementary Figure 7. UGT2B17 knockout altered gene transcription associated with DDR and cell mitosis
(A) RNA sequencing analysis comparing LNCaP95 and LN95(KO) cells was presented as a volcano plot ($P < 0.05$; fold change > 2). **(B)** LNCaP95 and LN95(KO) cells were treated with increasing doses of etoposide. Indicated proteins were measured by immunoblotting.

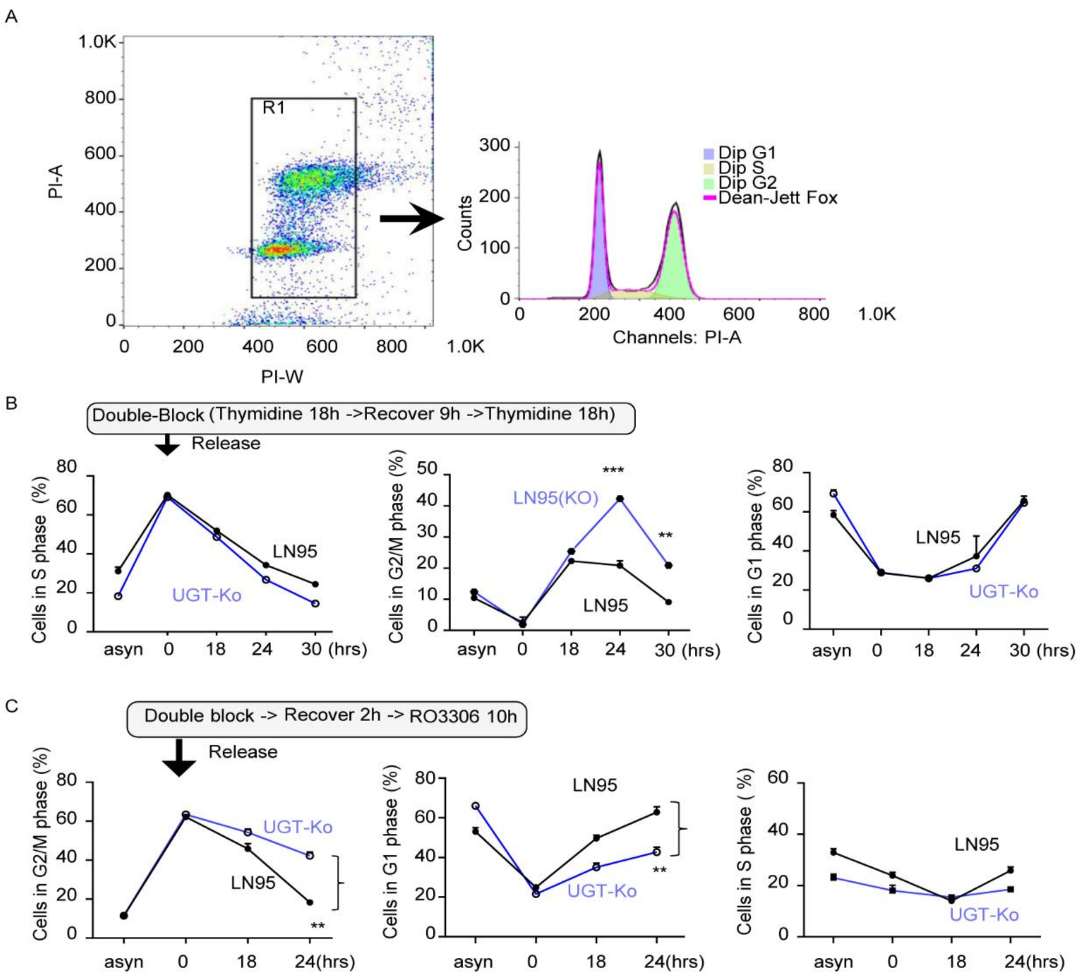

Supplementary Figure 8. UGT2B17 regulates gene expression in association with cell cycling

(A and B) LNCaP95 and LN95(KO) cells were synchronized using a double thymidine block and then released into growth medium. Flow cytometry analysis was performed to profile the cell populations in the G1, S, and G2/M phases of the cell cycle. **(C)** LNCaP95 and LN95(KO) cells were synchronized using a double thymidine block followed by RO3306 treatment and then released into growth medium. Flow cytometry analysis was performed to profile the cell populations in the G1, S, and G2/M phases of the cell cycle. Data are shown as the mean ± SEM. Statistical tests performed by two-way ANOVA test. ** $P < 0.01$.

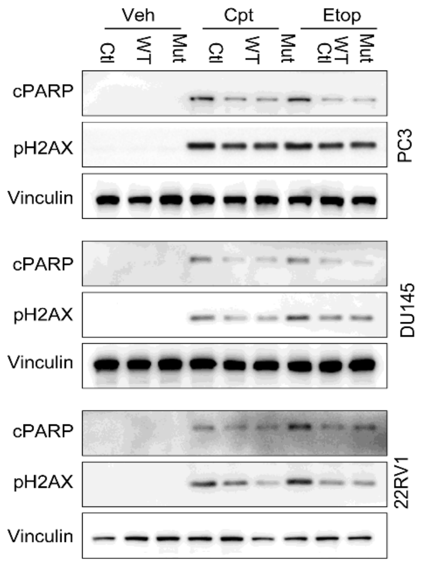

Supplementary Figure 9. UGT2B17 prevents PCa cells from undergoing apoptosis
PC3, Du145 and 22RV1 cells were transfected with control, UGT2B17 and UGTm for 72 hours, and then treated with 10uM etoposide or 1uM camptothecin for 24 hours. Cell lysates were collected for immunoblotting using indicated antibodies.

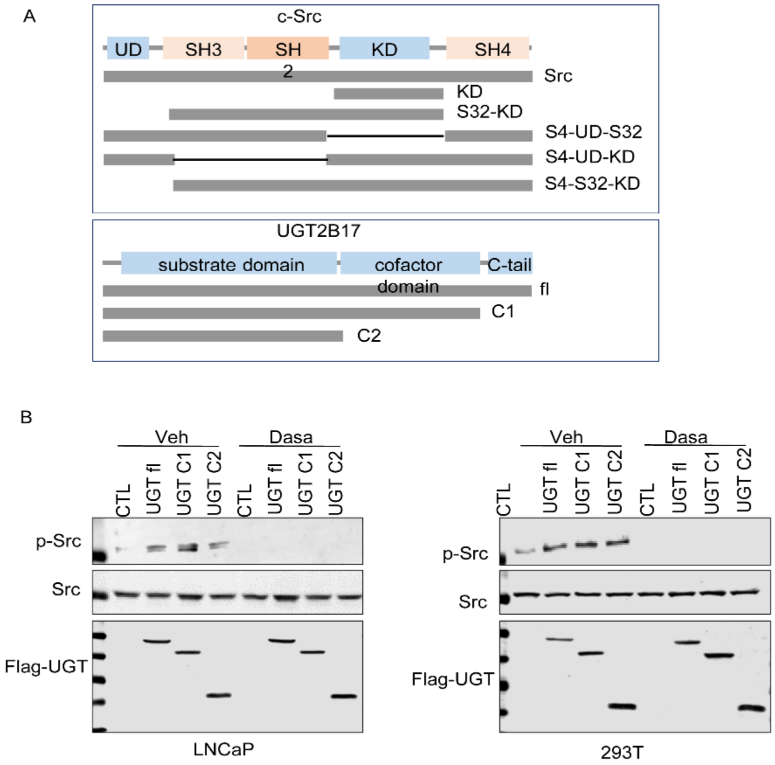

Supplementary Figure 10. Protein-protein interactions between UGT2B17 and Src
(A) Domain organization of Src and UGT2B17 proteins, along with their truncated mutants carrying various functional domains. **(B)** LNCaP and 293T cells were transfected with Flag-tagged UGT2B17 and its

mutants (C1, aa 1-438; C2, aa 1-256). Cells were then treated with either vehicle or 10 nM dasatinib for 1 hour. Src, p-Src(419), and Flag-UGT were detected by immunoblotting.

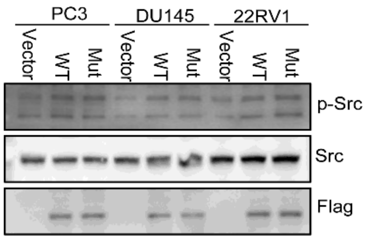

Supplementary Figure 11. UGT2B17 regulatesSRC activation

PC3, Du145 and 22RV1 cells were transfected with control, UGT2B17 and UGTm for 72 hours, and protein levels of total Src and p-Src(419) were measured by immunoblotting.

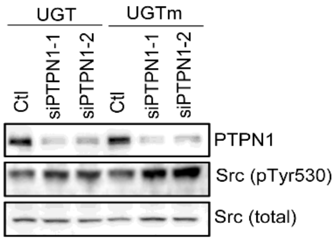

Supplementary Figure 12. The relationship between UGT2B17, PTPN1 and SRC

LN95(KO) cells were transfected with UGT2B17 or UGTm in the presence of control or siRNA against PTPN1, and protein levels of total Src and p-Src(530) were measured by immunoblotting.

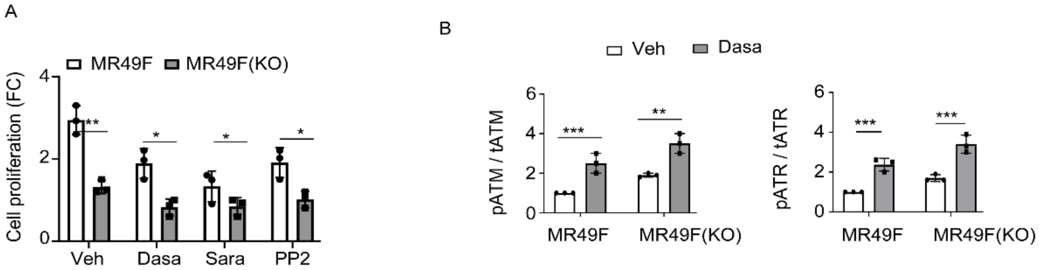

Supplementary Figure 13. The UGT2B17-Src axis regulates DDR

(A) MR49F and its UGT2B17 knockout cell line by CRISPR were treated with three Src inhibitors. Cell proliferation rates on day 3 were measured and normalized to that from MR49F cells at day 0. **(B)** MR49F and its UGT2B17 knockout cell line were treated with either vehicle or 10 nM dasatinib for 1 hour. ATM, ATR, and their phosphorylated forms were measured by immunoblotting. Densitometry of protein bands was calculated as the ratio of p-ATM and p-ATR to total ATM and ATR. Data are shown as the mean ± SEM. Statistical tests performed by two-way ANOVA test. * $P < 0.05$, ** $P < 0.01$, *** $P < 0.001$.

Table S1. Reagents

Reagent name	Source
Thapsigargin	Abcam, Cat #ab120286
PACMA31	MCE, Cat #HY-100433
MG132	Cayman, Cat #13697
Dasatinib(Dasa)	Cayman, Cat #11498
Saracatinib (Sara)	Cayman, Cat # 11497
PP2	Bio-techne, Cat #1407
Cycloheximide	Cayman, Cat # 66-81-9
Thymidine	Cayman, Cat #50-89-5
RO3306	Cayman, Cat #872573-93-8
Camptothecin(CPT)	Sigma, Cat # PHL89593
Etoposide	MCE, Cat # HY-13629
P4HB protein with His	TargetMol, Cat#TMPY-01012
PDIA3 protein with His	TargetMol, Cat#TMPJ-00935

Table S2. Antibodies

Antibody Target	Source	Application
UGT2B17(EL-95)	from Dr. Éric Lévesque	IHC
UGT2B17(EL-2B17)	From Dr. Éric Lévesque	IHC
SRC	Santa Cruz, Cat#sc-8056	co-IP, WB
pSRC(Tyr419)	Cell signaling technology, Cat#2101S	WB;IHC
pSRC(Tyr530)	Affinity, Cat#AF3161	WB
PTPN1	Proteintech, Cat# 11334-1-AP	WB
c-PARP	Cell signaling technology, Cat#9541S	WB
pH2AX(Ser139)	Millipore, Cat#JBW301	WB
pATM(S1981)	Abcam, Cat#ab81292	WB
pATR(T1989)	Abcam, Cat#223258	WB;IHC
ATM	Abcam, Cat#ab78	WB
ATR	Abcam, Cat#ab2905	WB
Calnexin	Abcam, Cat#ab133615	WB
ERP44	Proteintech, Cat# 16016-1-AP	WB
BiP	Proteintech, Cat# 11587-1-AP	WB
PDIA3	Abcam, Cat#ab154191	WB
SEL1L	Proteintech, Cat# 84317-7-RR	WB
GANAB	Proteintech, Cat# 83324-1-RR	WB
PERK	Cell signaling technology, Cat# 3192	WB
EIF2a	Cell signaling technology, Cat#5324	WB

pEIF2a	Cell signaling technology, Cat#3398	WB
CHOP	Proteintech, Cat# 15204-1-AP	WB
PDIA1	Abcam, Cat#ab 137110	WB
Actin	Santa Cruz, Cat# sc-1615	WB
Vinculin	Sigma, Cat#V9131	WB
Tubulin	Abcam, Cat#ab18251	WB
LaminA/C	Santa Cruz, Cat# sc- 7293	WB
Flag	Cell signaling technology, Cat#14793	co-IP, WB
Flag	Abmart, Cat#M20008	IF
His	Proteintech, Cat# 66005-1-Ig	co-IP, WB
HA	Cell signaling technology, Cat# 3724S	co-IP, WB

Table S3. Primers

Gene Name	Sequence (5´to 3´)
GAPDH	F: AGC CTG GAC AAA TGG CAT TCA
	R: GGA CCT GAC CTG CCG TCT AGA A
CCNA1	F: CGC TGG CGG TAC TGA AGT C
	R: AAG GAG GAA CGG TGA CAT GC
CCNA2	F: TTCACACATACCTTAGGGAAATGG
	R: AGCCAAATGCAGGGTCTCAT
CCNB1	F: ATA AGG CGA AGA TCA ACA TGG C
	R: TTT GTT ACC AAT GTC CCC AAG AG
CCNB2	F: GCGTTGGCATTATGGATCG
	R: TCTTCCGGGAAACTGGCTG
CCND1	F: GTG CTG CGA AGT GGA AAC C
	R: ATC CAG GTG GCG ACG ATC T
AURKB	F: CGCAGAGAGATCGAAATCCAG
	R: AGATCCTCCTCCGGTCATAAAA
UBE2C	F: GAC CTG AGG TAT AAG CTC TCG C
	R: TTA CCC TGG GTG TCC ACG TT
CDK1	F: GGA AAC CAG GAA GCC TAG CAT C
	R: GGA TGA TTC AGT GCC ATT TTG CC
CHOP	F: CAGAACCAGCAGAGGTCACA
	R: AGCTGTGCCACTTTCCTTTC

GADD34	F: TCCGACTGCAAAGGCGGCTCA
	R: CAGCCAGGAAATGGACAGTGAC
NOXA	F: TTTCTTCGGTCACTACACAACG
	R: GAGCATTTTCCGAACCTTTAGA
XBP1s	F: TAAGACAGCGCTTGGGGATG
	R: GCACGTAGTCTGAGTCGTGC
PRKDC	F: AGCTGGCTTGCGCCTATTT
	R: GGGCACACCACTTTAACAAGA
FANC1	F: TTCTCACTGCTCTTTTCAGGGAT
	R: GCCCTGTTTCCTTTAGCTGC
BRCA1	F: GAAACCGTGCCAAAAGACTTC
	R: CCAAGGTTAGAGAGTTGGACAC
BRCA2	F: CACCCACCCTTAGTTCTACTGT
	R: CCAATGTGGTCTTTGCAGCTAT
PARP2	F: GCCTTGCTGTTAAAGGGCAAA
	R: TCCTTCACAATACACATGAGCC
XRCC3	F: ATGGATTTGGATCTACTGGACCT
	R: CTTCCCCGCAAGTGTAAGGAG
XRCC2	F: TGCTTTATCACCTAACAGCACG
	R: TGCTCAAGAATTGTAACTAGCCG
CHEK1	F: ATATGAAGCGTGCCGTAGACT
	R: TGCCTATGTCTGGCTCTATTCTG
PDIA4	F: AGTGGGGAGGATGTCAATGC
	R: TGGCTGGGATTTGATGACTG
SEL1L	F: ATCTCCAAAAGGCAGCAAGC
	R: TGGGAGAGCCTTCCTCAGTC
HYOU1	F: GCAGACCTGTTGGCACTGAG
	R: TCACGATCACCGGTGTTTTC
EDEM1	F: TTCCCTCCTGGTGGAATTTG
	R: AGGCCACTCTGCTTTCCAAC
