# Supplementary material for: Noncanonical functions of UGT2B17 promote castration-resistant prostate cancer progression
Source: J Clin Invest. 2025 Dec 4;136(2):e196495. doi: 10.1172/JCI196495 (PMC12807465; doi:10.1172/JCI196495)

Full unedited blot/gel for Figure 2C

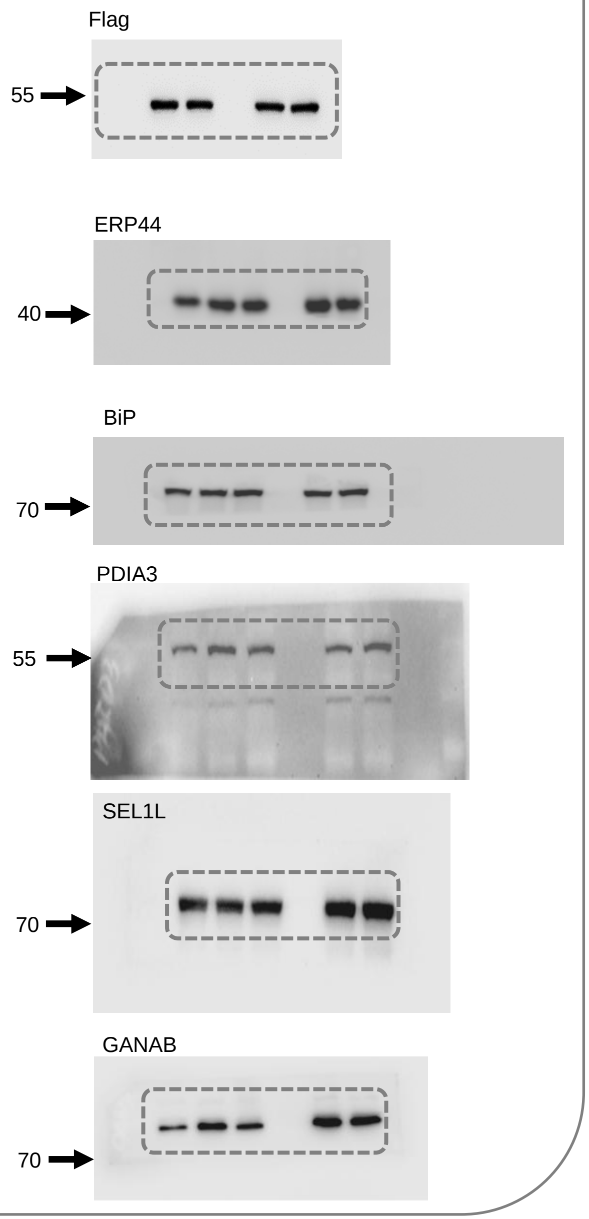

Full unedited blot/gel for Figure 2G

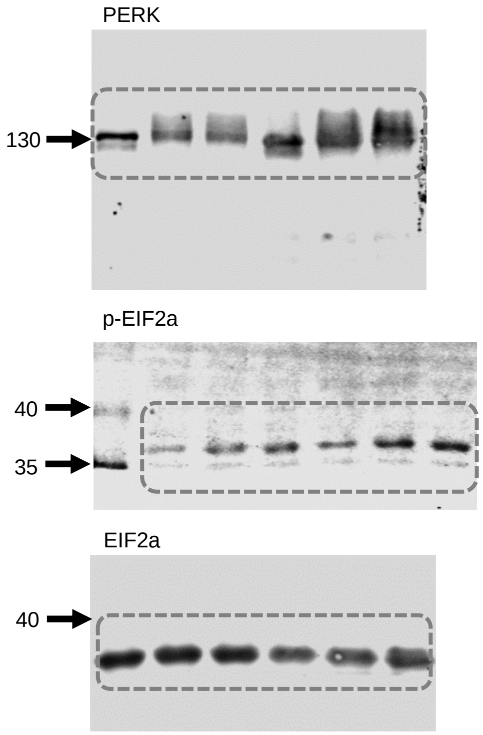

Full unedited blot/gel for Figure 3F

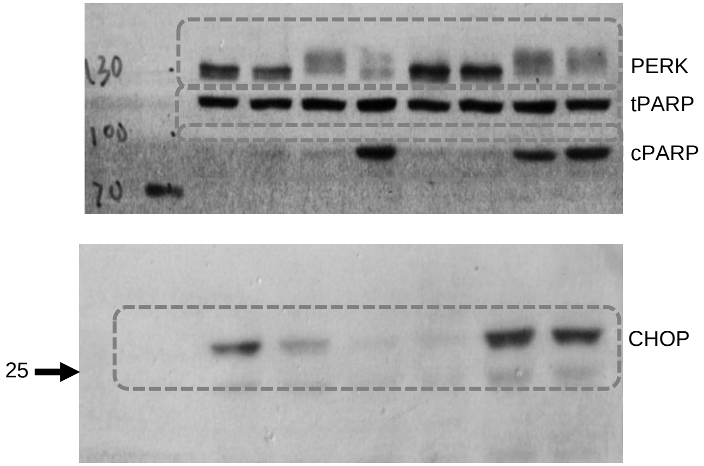

Full unedited blot/gel for Figure 3H

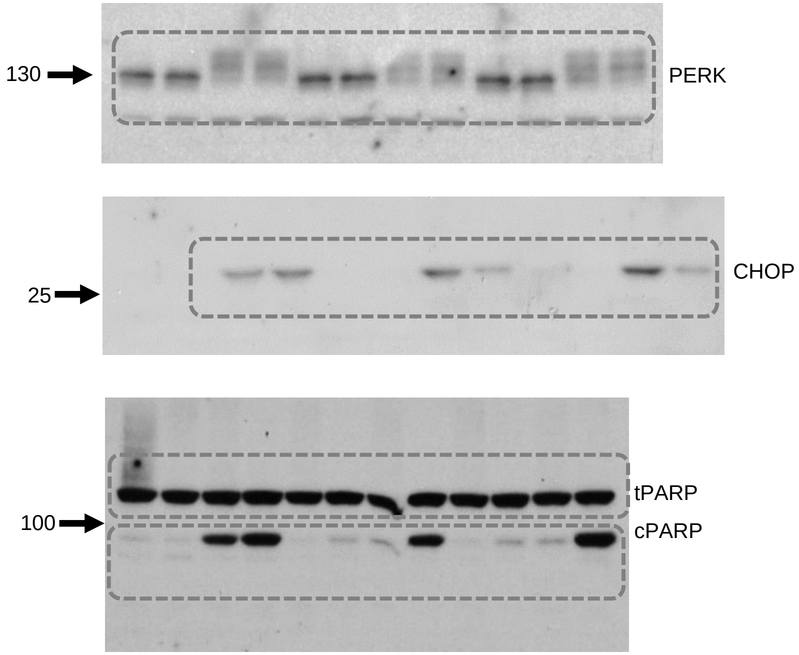

Full unedited blot/gel for Figure 4D

cPARP

100

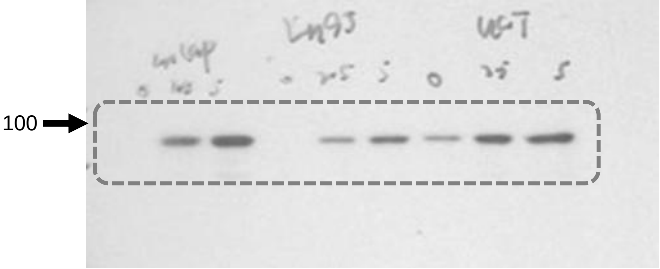

pH2AX

15

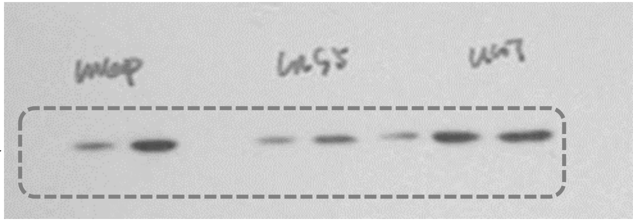

Vinculin

100

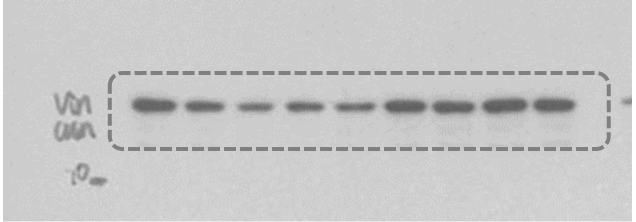

Full unedited blot/gel for Figure 4H

cPARP

100

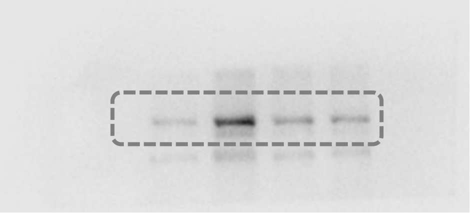

Tubulin

55

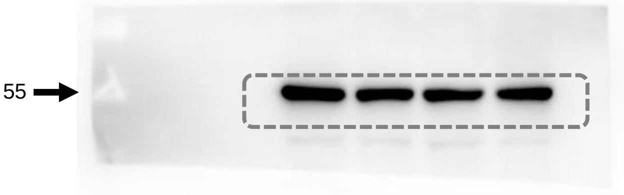

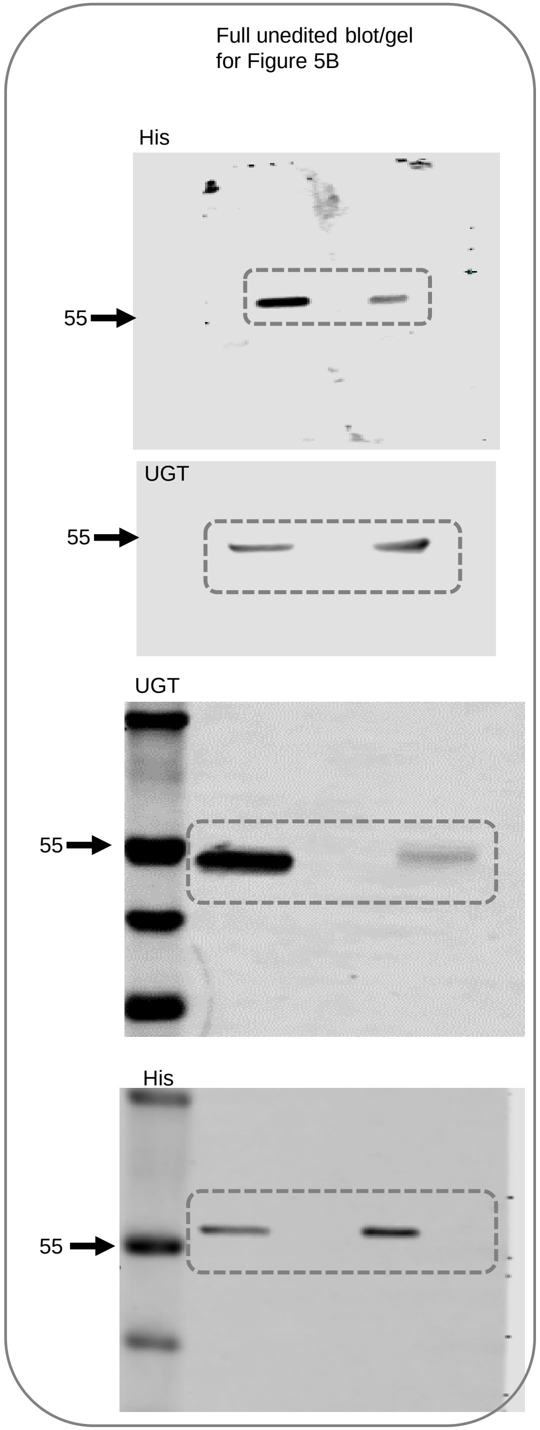
Full unedited blot/gel
for Figure 5B
His
55
UGT
55
UGT
55
His
55

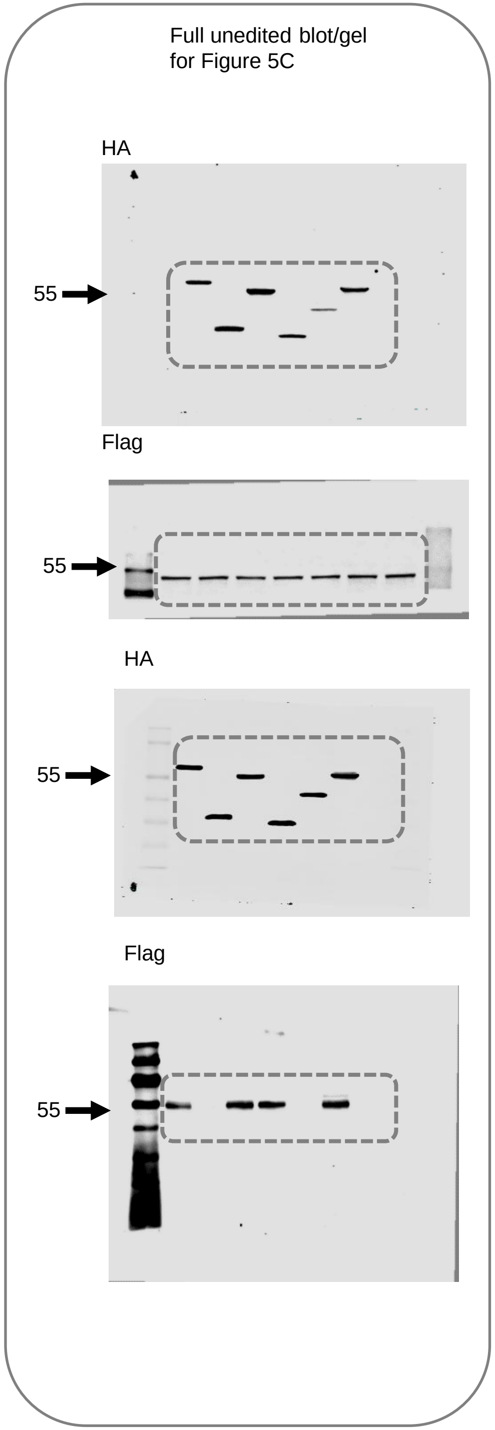
Full unedited blot/gel
for Figure 5C
HA
55
Flag
55
HA
55
Flag
55

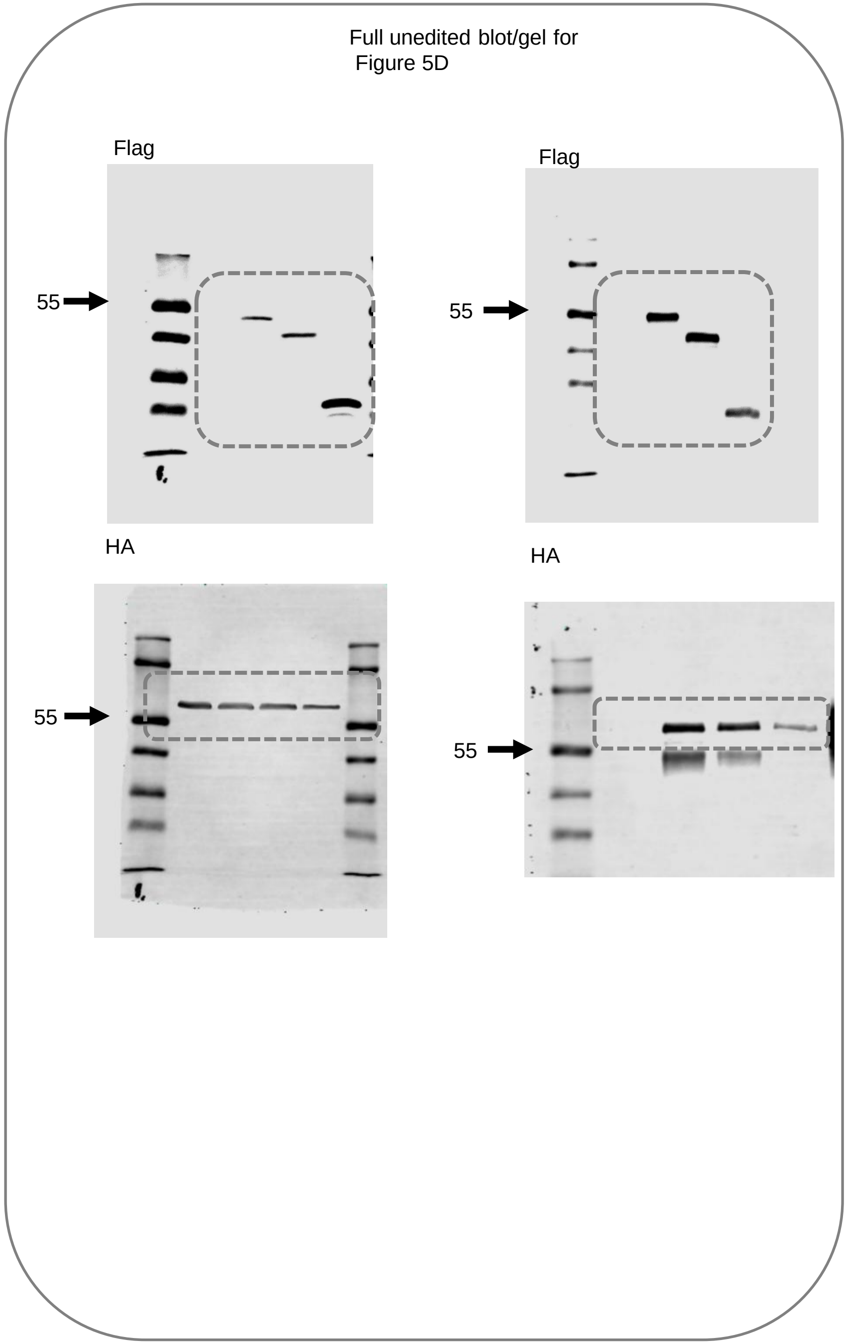
Full unedited blot/gel for
Figure 5D
Flag
55
Flag
55
HA
55
HA
55

Full unedited blot/gel
for Figure 5E

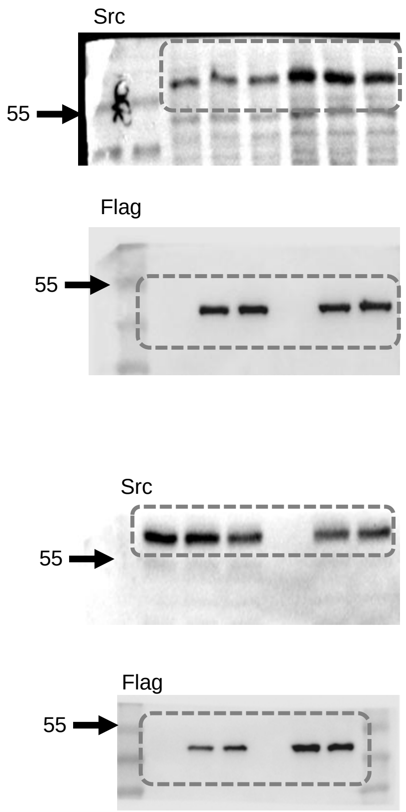

Full unedited blot/gel
for Figure 5F

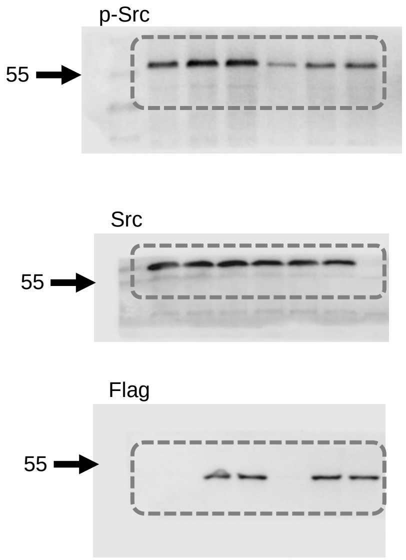

Full unedited blot/gel
for Figure 5G

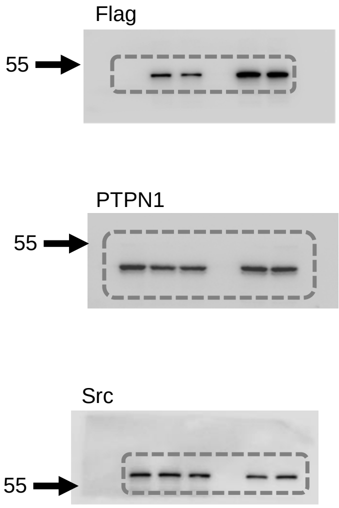

Full unedited blot/gel
for Figure 5H

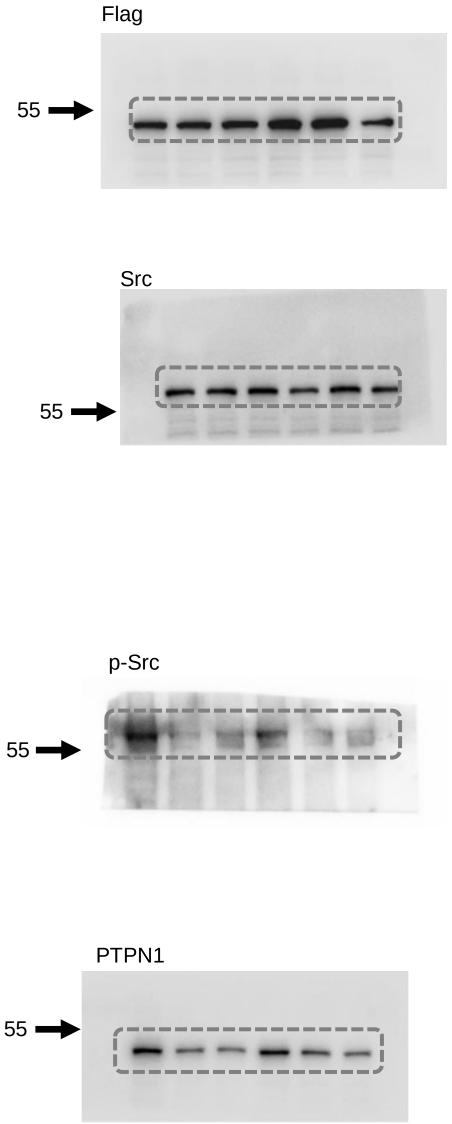

Full unedited blot/gel for Figure 6I

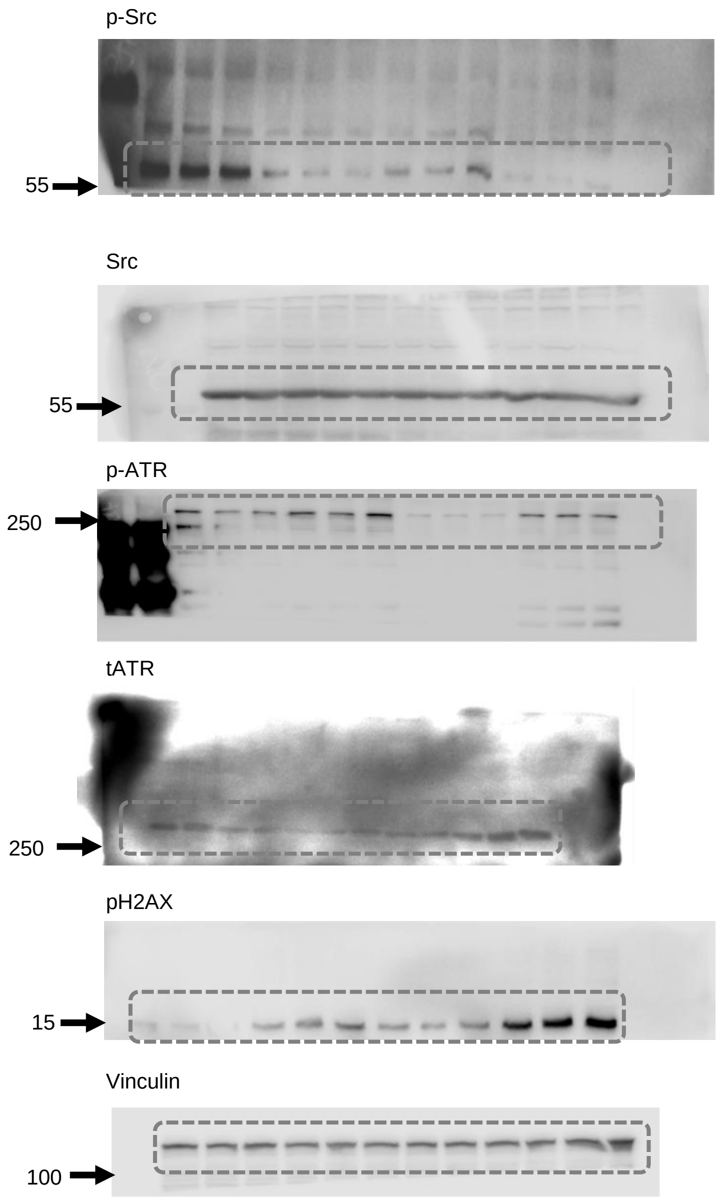

Full unedited blot/gel for Figure S2D

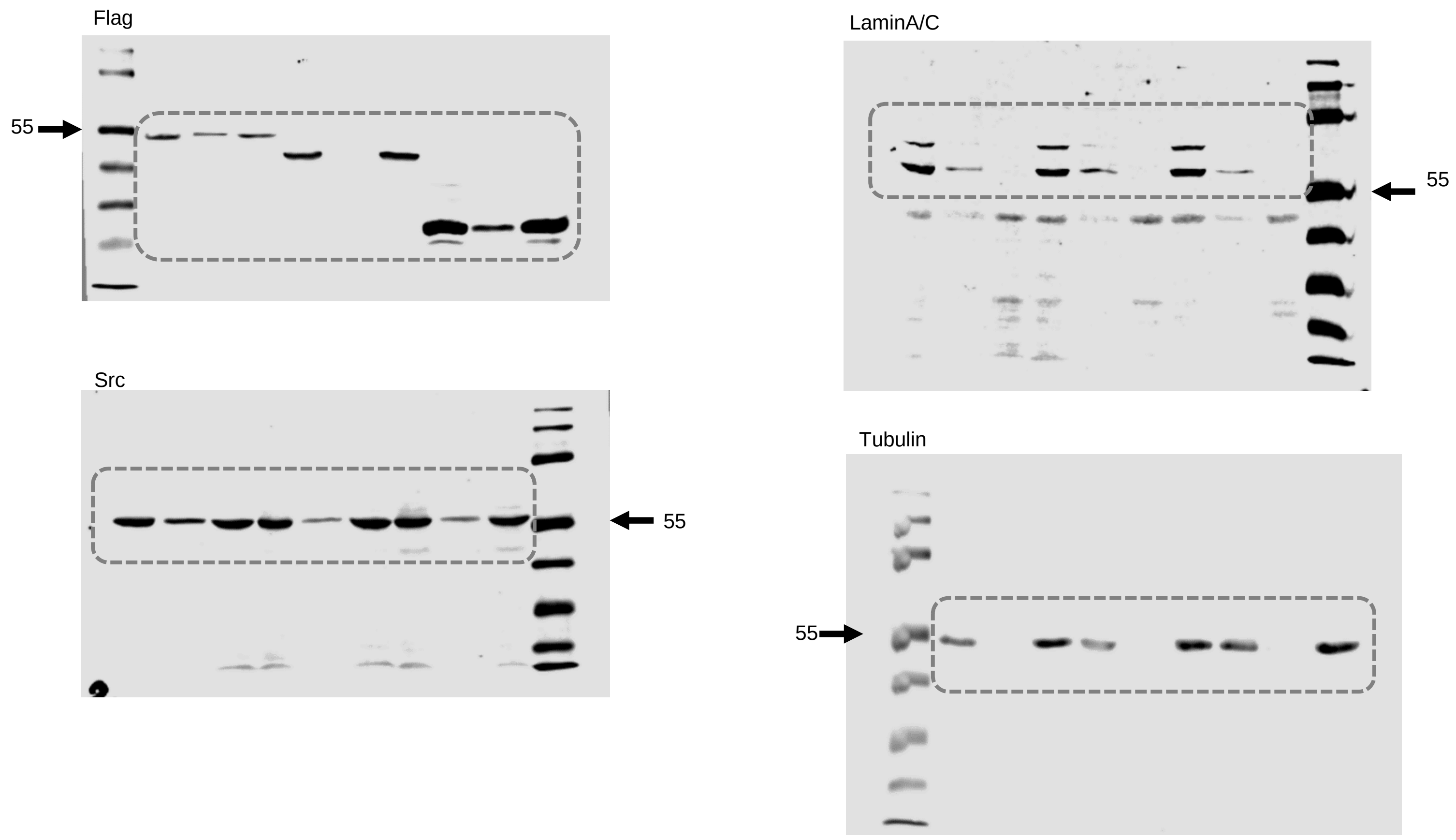

Full unedited blot/gel
for Figure S3A

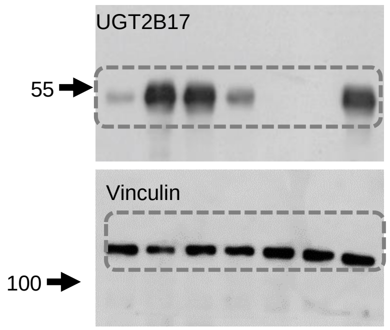

Full unedited blot/gel
for Figure S3B

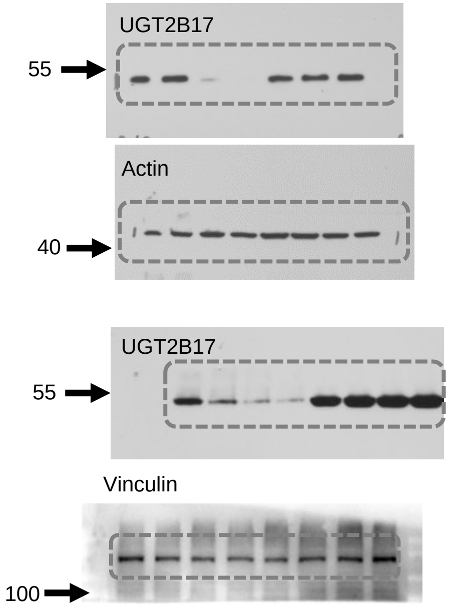

Full unedited blot/gel
for Figure S3C

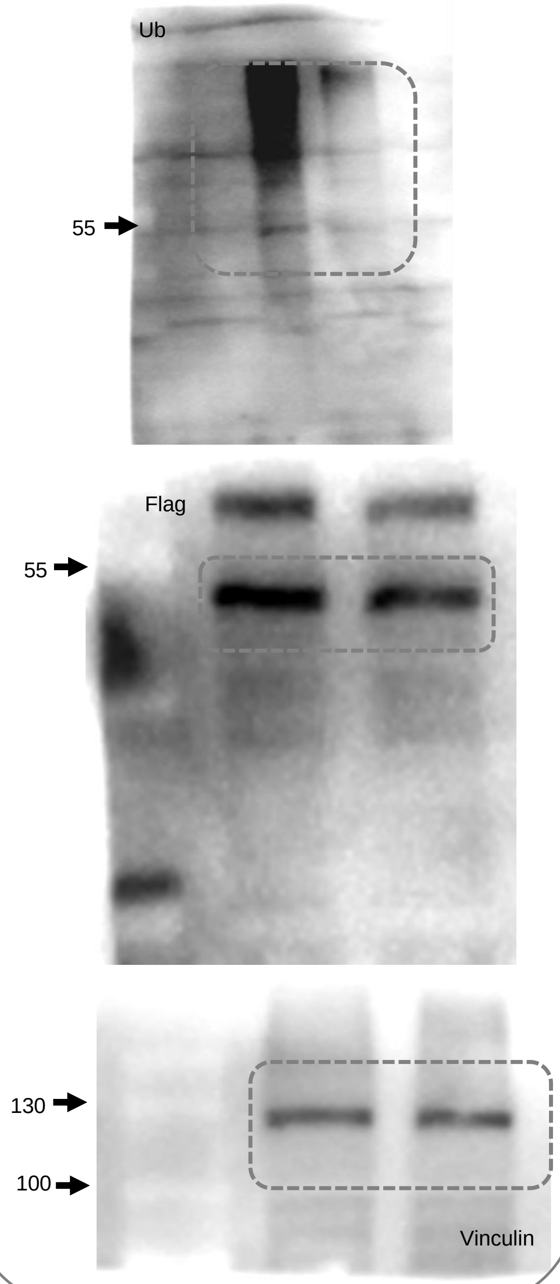

Full unedited blot/gel
for Figure S3E

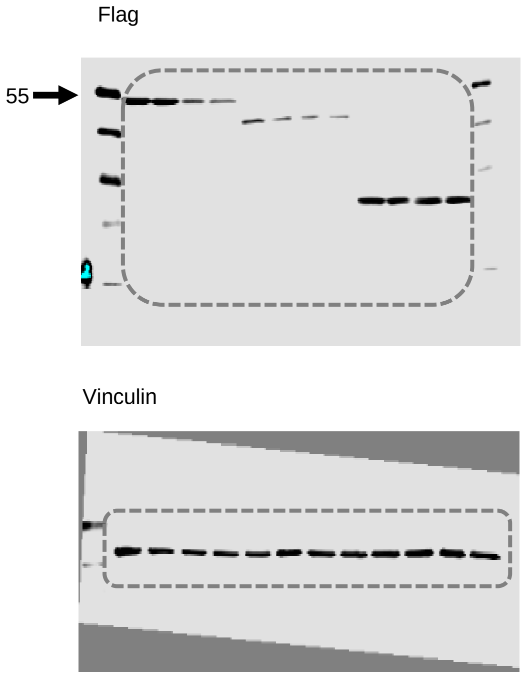

Full unedited blot/gel for Figure S4A

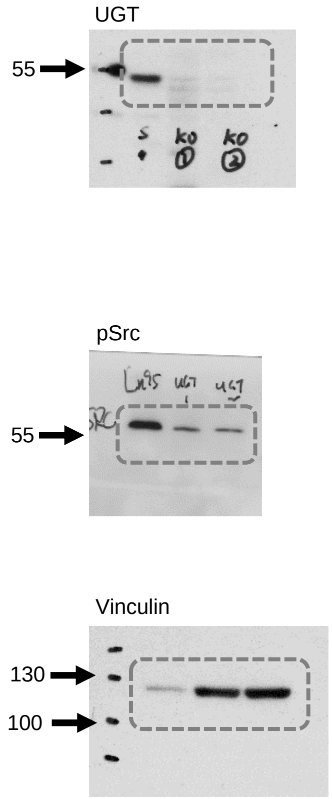

Full unedited blot/gel for Figure S6B

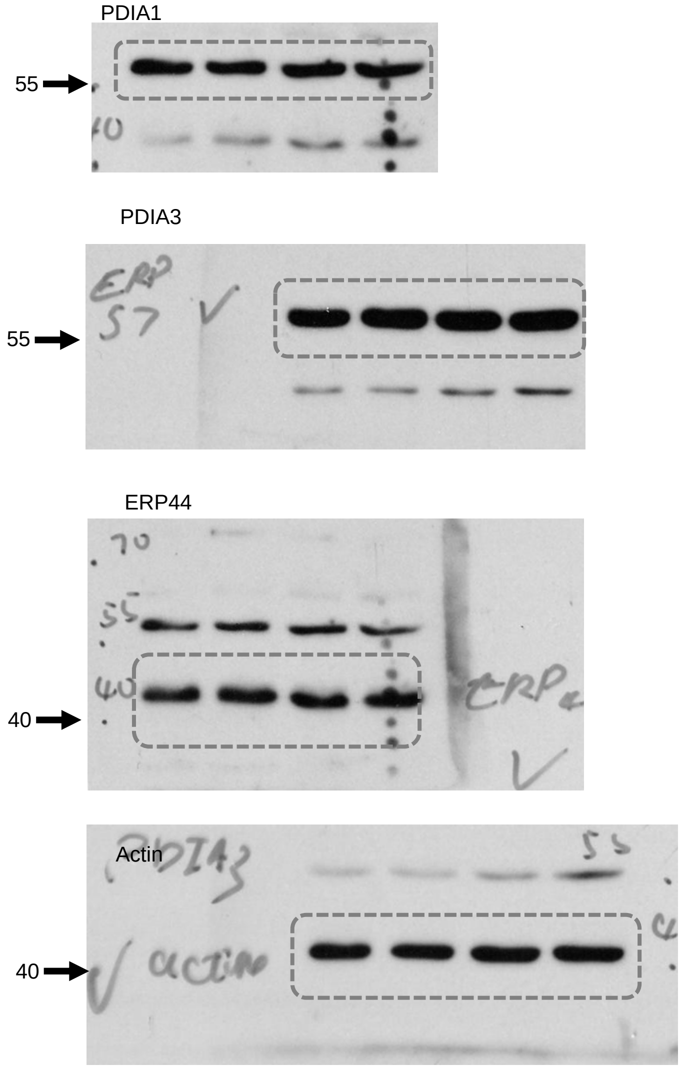

Full unedited blot/gel for Figure S6C

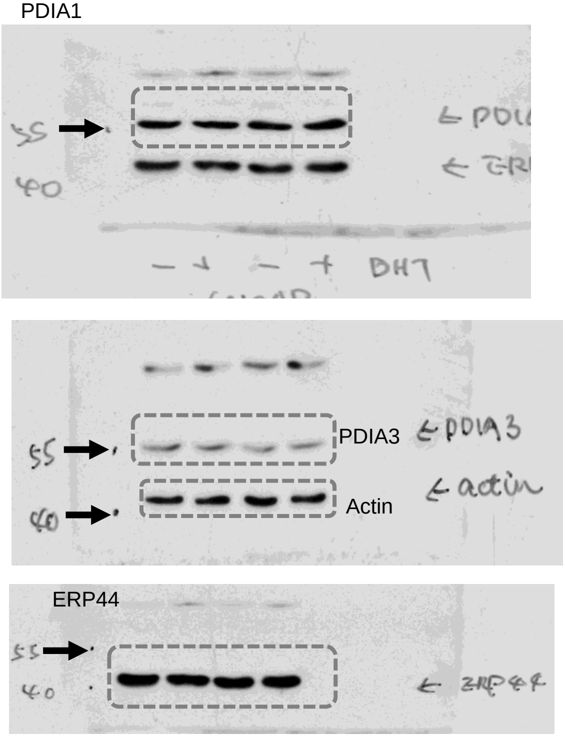

Full unedited blot/gel
for Figure S6D

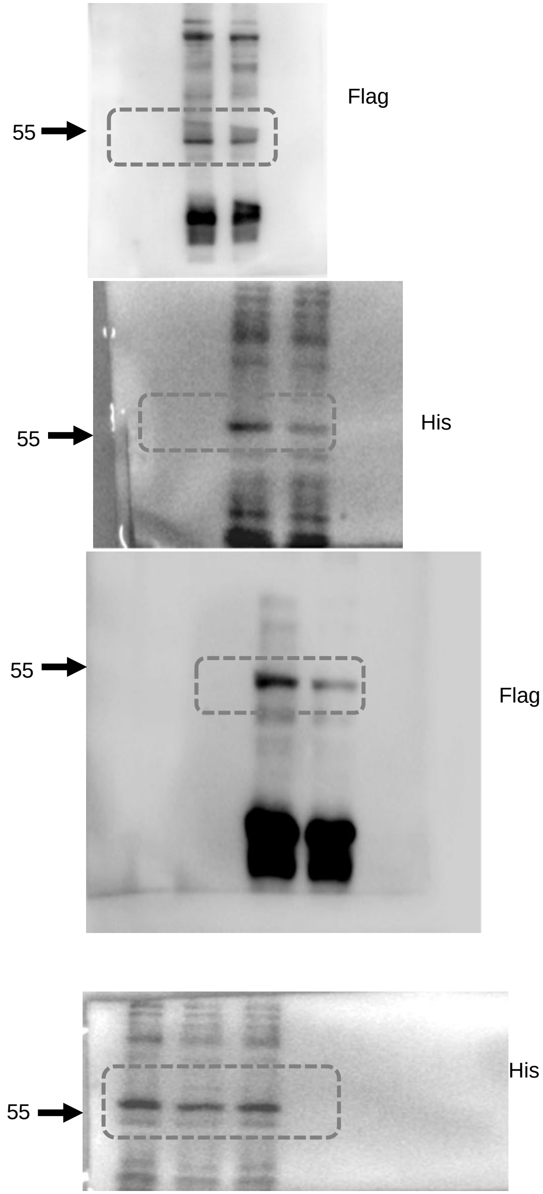

Full unedited blot/gel
for Figure S6E

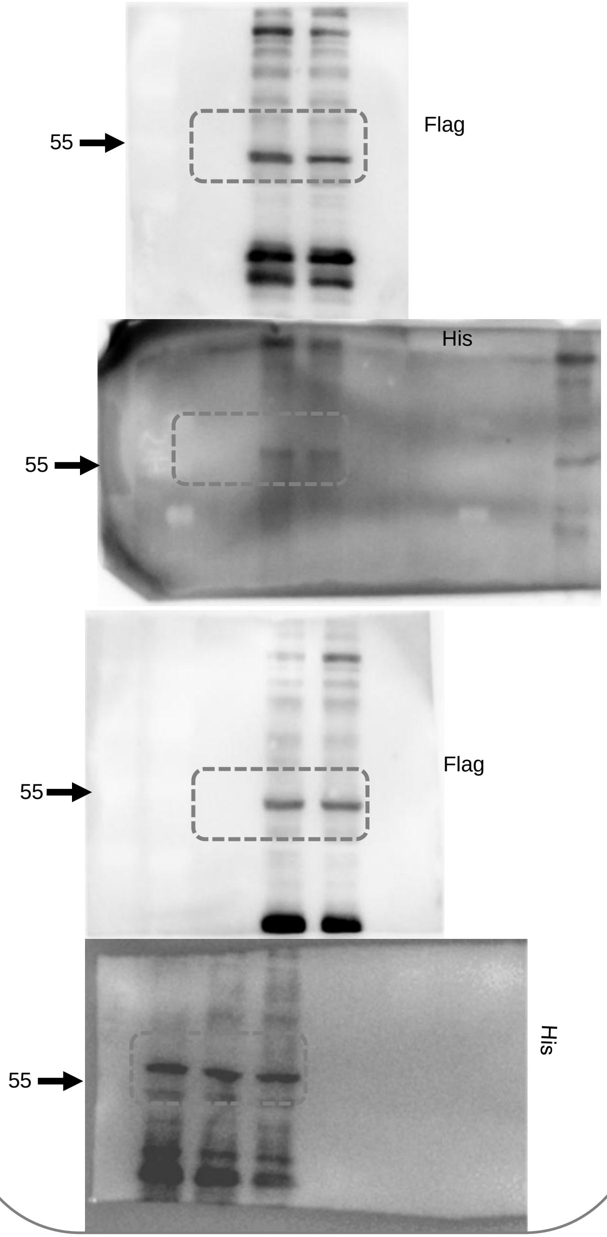

Full unedited blot/gel
for Figure S7B

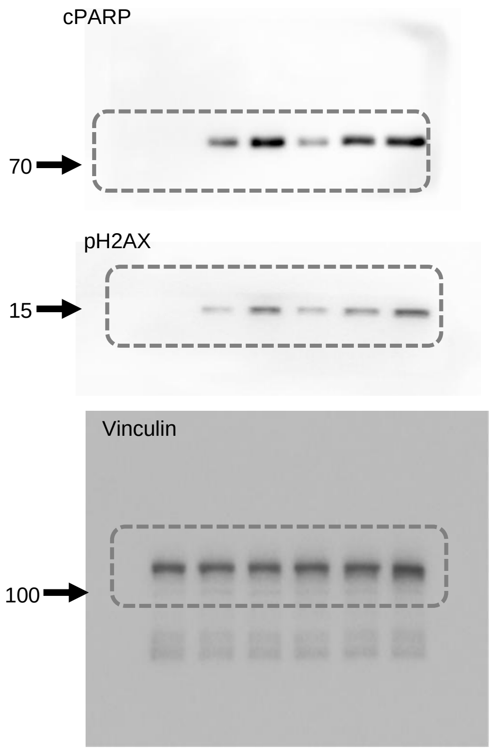

Full unedited blot/gel for Figure S9

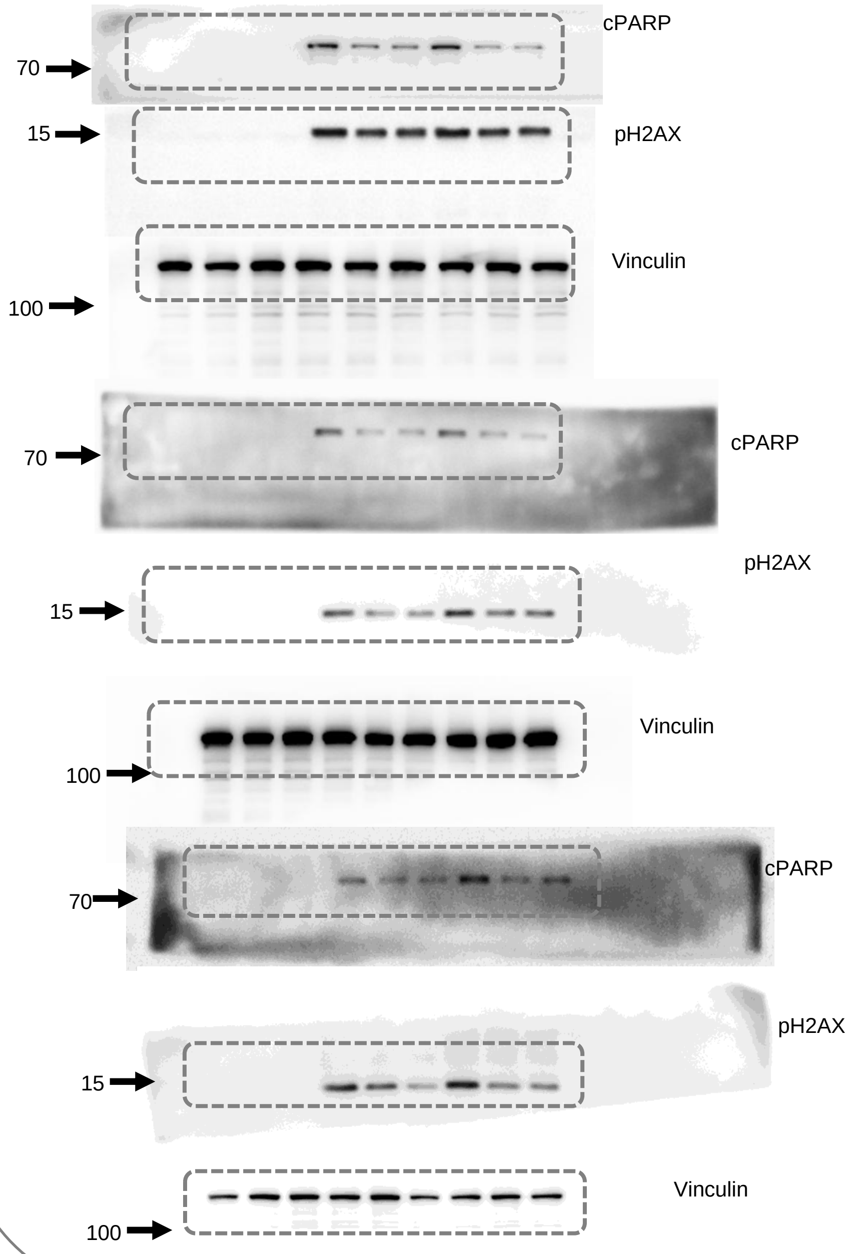

Full unedited blot/gel for Figure S10B

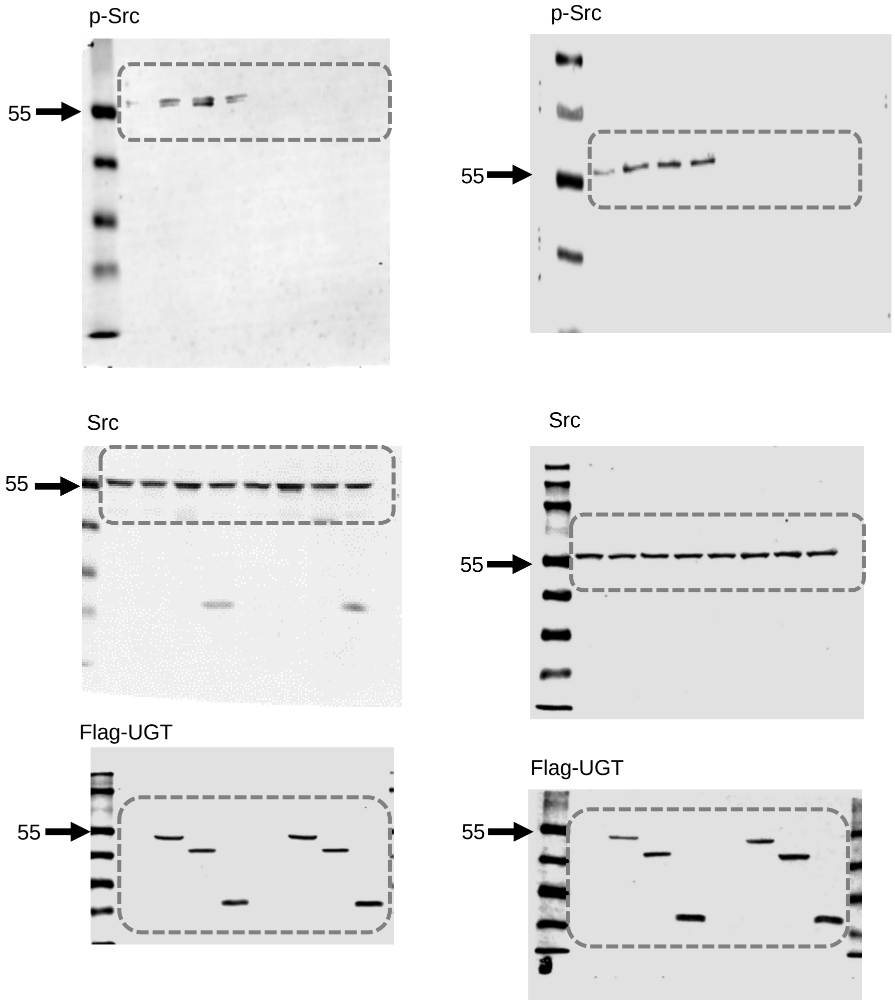

Full unedited blot/gel
for Figure S11

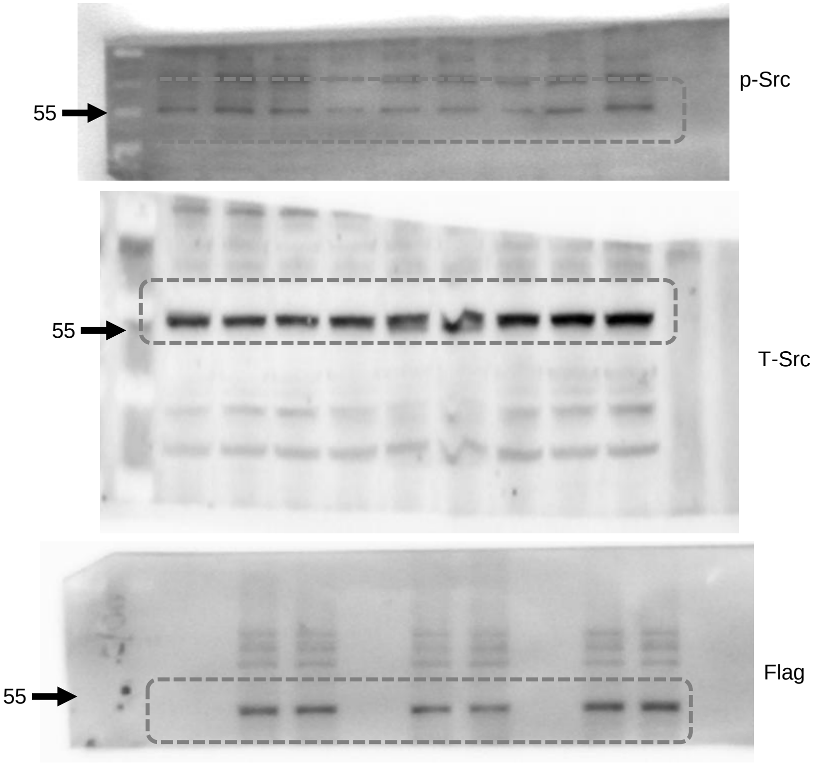

Full unedited blot/gel
for Figure S12

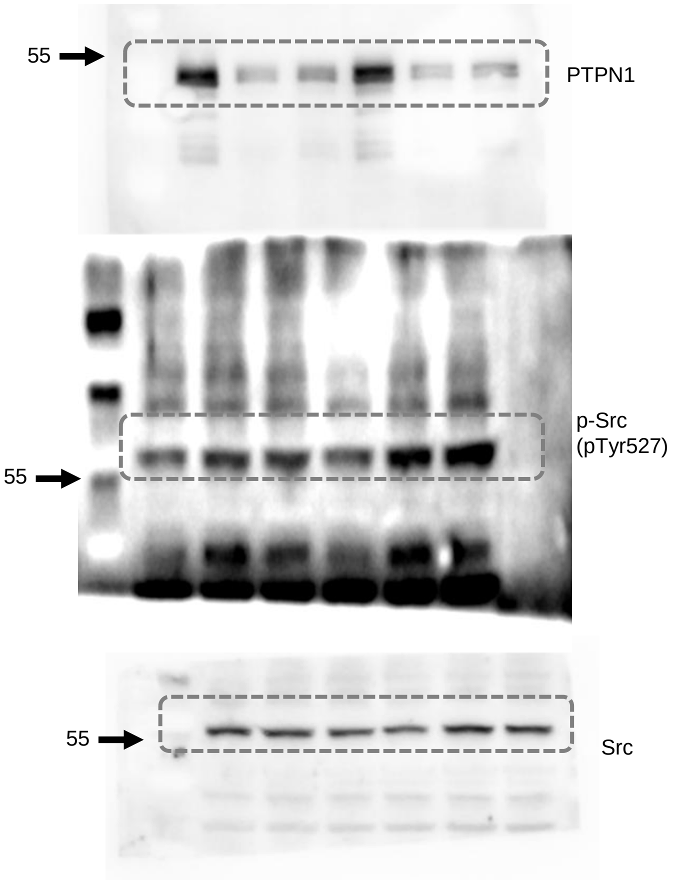

Supplement: Unedited blot and gel images [file jci-136-196495-s120.pdf]
